# Bell correlations outside physics

**DOI:** 10.1038/s41598-023-31441-x

**Published:** 2023-03-16

**Authors:** C. Gallus, E. M. Pothos, P. Blasiak, J. M. Yearsley, B. W. Wojciechowski

**Affiliations:** 1 Technische Hochschule Mittelhessen, 35390 Gießen, Germany; 2 City, University of London, London, EC1V 0HB UK; 3 Institute for Quantum Studies, Chapman University, Orange, CA USA; 4 Institute of Nuclear Physics Polish Academy of Sciences, 31342 Kraków, Poland; 5 Institute of Applied Psychology, Jagiellonian University, 30348 Kraków, Poland

**Keywords:** Applied physics, Quantum physics, Statistical physics, thermodynamics and nonlinear dynamics

## Abstract

Correlations are ubiquitous in nature and their principled study is of paramount importance in scientific development. The seminal contributions from John Bell offer a framework for analyzing the correlations between the components of quantum mechanical systems and have instigated an experimental tradition which has recently culminated with the Nobel Prize in Physics (2022). In physics, Bell’s framework allows the demonstration of the non-classical nature of quantum systems just from the analysis of the observed correlation patterns. Bell’s ideas need not be restricted to physics. Our contribution is to show an example of a Bell approach, based on the insight that correlations can be broken down into a part due to common, ostensibly significant causes, and a part due to noise. We employ data from finance (price changes of securities) as an example to demonstrate our approach, highlighting several general applications: first, we demonstrate a new measure of association, informed by the assumed causal relationship between variables. Second, our framework can lead to streamlined Bell-type tests of widely employed models of association, which are in principle applicable to any discipline. In the area of finance, such models of association are Factor Models and the bivariate Gaussian model. Overall, we show that Bell’s approach and the models we consider are applicable as general statistical techniques, without any domain specificity. We hope that our work will pave the way for extending our general understanding for how the structure of associations can be analyzed.

## Introduction

The understanding of correlations is crucial for theoretical progress throughout science. For example, in psychology, formal analysis of social networks often quantifies different kinds of interaction between agents in terms of correlation functions (e.g., in this journal,^1^). In zoology, it might be of interest to study how correlations in the behavior between the organisms comprising an ecosystem varies with environmental characteristics, such as rainfall. In epidemiology, correlation is often the basis for attempts to understand the causal drivers of changes in the spread of particular diseases (e.g., in this journal,^2^). In engineering, correlations between the variables characterizing a complex system, such as the temperature of different components, might shed light on the properties of the system or help with troubleshooting. In economic theory, positive correlation between performance outcomes might signal competition^3^. In this journal^4^, complex financial systems have been studied in terms of interaction mechanisms ultimately based on correlation.

It hardly seems necessary to motivate the importance of studying correlation structure across science. Yet, there has been a hugely influential approach to correlation in physics, with so far negligible impact in the rest of science. John Bell developed what is arguably the most sophisticated framework for correlations in physics, showing how certain assumptions about the structure of causal relationships between two pairs of variables produce a distinctive signature on the observed correlations. In particular, certain natural assumptions about the causes of physical phenomena lead to the so-called Bell inequality. The point of Bell’s analysis was to argue that, if using a causal analysis we can exclude any ’classical’ influences on any observed correlations, then particular correlation patterns could only be explained by something not classical in the physical nature of the corresponding systems - this has been the essential argument for how a Bell test can be used as evidence for the non-classical nature of quantum structure in the physical world. Bell’s work has inspired the exciting experiments conducted by Aspect, Clauser, and Zeilinger, whose importance has been recognized with the recent Nobel Prize in Physics (2022).

At this point, we have to offer a disclaimer to our readers: our work is intended to be of general interest, concerning any situation where there is a need to understand the structure of correlations. However, the bulk of work concerning Bell’s framework has been conducted in physics. Therefore, much of the ensuing discussion inevitably borrows from corresponding work in physics and extends this work accordingly.

In physics, the derivation of the inequalities in a Bell experiment rests on the assumptions of realism, locality and free choice, while any observed violations show that models insisting on all three assumptions run into contradictions with physical reality. Bell experiments are performed by subjecting two space-time separated components of an entangled particle system to certain measurements (e.g. spin measurements). Note that different notions of non-locality exist, for example those based on information retrieval and local state discrimination^5^, whereas the perspective taken here is based on causal mechanisms. The experimenters on each side choose the regimes *x*, *y* freely from one of two possible spin directions. The result of the two measurements are recorded as *a* and *b*, respectively. A time series of quadruplets (*a*, *b*, *x*, *y*) results, from which a statistic $P( ab \, | \, xy )$ and four expectation values $\langle ab\rangle _{\scriptscriptstyle xy}=\sum _{\scriptscriptstyle a,b}\,ab\,P( ab \, | \, xy )$ can be computed. For simplicity we use the compact notation $\sum _{a,b} ab\,P(ab|xy)$ when we mean $\sum _{a,b} ab\,P(a,b|x,y)$.

The combination of these four expectation values yields the four *S*-values

1 $$\begin{aligned} S_{\scriptscriptstyle 1}= & {} \ \ \ \langle ab\rangle _{\scriptscriptstyle 00}+\langle ab\rangle _{\scriptscriptstyle 01}+\langle ab\rangle _{\scriptscriptstyle 10}-\langle ab\rangle _{\scriptscriptstyle 11}, \end{aligned}$$

2 $$\begin{aligned} S_{\scriptscriptstyle 2}= & {} \ \ \ \langle ab\rangle _{\scriptscriptstyle 00}+\langle ab\rangle _{\scriptscriptstyle 01}-\langle ab\rangle _{\scriptscriptstyle 10}+\langle ab\rangle _{\scriptscriptstyle 11}, \end{aligned}$$

3 $$\begin{aligned} S_{\scriptscriptstyle 3}= & {} \ \ \ \langle ab\rangle _{\scriptscriptstyle 00}-\langle ab\rangle _{\scriptscriptstyle 01}+\langle ab\rangle _{\scriptscriptstyle 10}+\langle ab\rangle _{\scriptscriptstyle 11}, \end{aligned}$$

4 $$\begin{aligned} S_{\scriptscriptstyle 4}= & {} \ -\langle ab\rangle _{\scriptscriptstyle 00}+\langle ab\rangle _{\scriptscriptstyle 01}+\langle ab\rangle _{\scriptscriptstyle 10}+\langle ab\rangle _{\scriptscriptstyle 11}. \end{aligned}$$

Note, here we follow the Clauser-Horne-Shimony-Holt (CHSH) approach^6^, as their variant of the original Bell inequalities are slightly simpler and, in any case, better suited to the present purposes. Either way, this provides us with a tool to make testable distinctions between different causal models for a given dataset. Specifically, Bell’s seminal ideas lead to the conclusion that any realist local hidden variable model where experimenters can freely chose *x*, *y* has to satisfy the following four inequalities

5 $$\begin{aligned} |S_{\scriptscriptstyle i}|\le 2&\qquad \text {for}\ \ i=1,\ldots \,,4. \end{aligned}$$

While the theoretical maximum value for the *S*-values is 4, an intuitive class of classical models leads to a maximum value of 2, whereas quantum mechanics predicts violations of that maximum, but only allows *S*-values up to the famous Tsirelson bound of $2\sqrt{2}$,^7^. Let us call the quantities from any of these equations *S*-values.

Depending on the experimental context and the causal model, violations of the Bell Inequalities (5) have sharply contrasting meaning. In realist models for quantum physics, they may be interpreted as violations of free choice or as violations of Bell locality^8,9^, or even as indications of retrocausality^10,11^.

In general, it will always be a challenge in extending a tool developed in physics, to the study of systems outside physics^12–14^. There are two difficulties in extending Bell’s framework to the study of correlations outside physics. First, the assumptions of locality and free choice in Bell’s framework are very particular to physics. Beyond the question of whether microscopic physical systems have quantum structure or not, locality and free choice have extremely limited interest. However, this difficulty does not pose a serious problem in putative extensions, since it is straightforward to imagine how analogous assumptions could guide suitable causal analyses in different situations. Amongst others, Pearl^15–17^ pioneered a formal methodology for doing so, aiming at the development of a theory of causal and counterfactual inference. Second, and perhaps more seriously, Bell’s framework involves two systems with two pairs of binary variables characterizing each system. The fact that we have a pair of binary variable pairs limits applicability outside physics, at least insofar as the study of correlation is concerned. This is because, in general, we are interested in the association between pairs of variables and, also, it is more practical to consider pairs. Outside the study of quantum mechanics, there are relatively few cases whereby a system is naturally characterized by a pair of variables, let alone binary ones. Indeed, existing applications of Bell inequalities outside physics often involve somewhat artificial set-ups for how to arrange variables so that Bell tests are possible (e.g., in behavioral sciences^18–20^).

Any general statistical measure inevitably simplifies situations, which are probably very complex. The correlation is a great example, insofar that the association between two variables is reduced to a single, linear index, regardless of any information about the causal processes linking the two variables. In seeking to apply Bell’s ideas outside physics, our aim is to develop an association index with some sensitivity to the causal structure relevant to two variables, but in a way which is as domain general as possible. The key assumption is that it is possible to separate the relatedness between two variables into two distinct parts, a part due to significant causes and a part due to incidental noise, and that the two parts can be distinguished in terms of the magnitude of variable change, at different parts of the variable’s range. We will see shortly how this assumption can be developed to a quantitative, precise framework.

In the remainder of the paper, we discuss a concrete application of these ideas, based on associations between the price change of different securities, in the S&P 500 index. There are three main reasons why we have chosen finance as an area for a first application of our framework. First, there is an immediately available, large data set. In the S&P 500 index, the information to construct variables corresponding to price changes for different securities within a temporal window is readily available: the S&P 500 offers 125,000 pairs of securities, against which we can test our new proposal for association, against standard correlation. Second, in finance, correlations play an important role. Correlations between the price change of different securities are key in creating optimal portfolios using Markowitz’s mean-variance model, while correlations between single securities and a broad market index enter the capital asset pricing model via the market beta and, from there, the valuation of companies via the discounted cash flow model^21–27^. Understanding the generative processes leading to correlations in the stock market is clearly a hugely involved task^28–37^. So, a key objective is whether the use of *S*-values, instead of correlations, affords any advantages. Finally, there have been several proposals aimed at capturing association structure in more detail. It is important to note that, even though the models we will discuss have their origins in finance research, they are general statistical models and can be applied in any area where there is a need for detailed understanding between variables.

In what follows, we first describe how the *S*-test in physics can be translated to something interesting in other areas. As noted, most of the mathematical methods follow from the Bell literature in physics. However, we intend our conclusions and analytical tools to be applicable in any area where there is a need to understand association structure in some detail, with finance being our chosen area of application presently.

### From physics to other disciplines

In physics experiments, precise assumptions about the structure of the systems under study enable detailed predictions concerning the ensuing correlations. Outside physics, such detailed assumptions and predictions are not possible in general terms. Indeed, the precise causal origins of some observed correlations are likely to vary across different areas of application. Nonetheless, a generic approach can be developed, by partitioning the relevant variable, for example in finance, *price change*, into different regimes, for example, into weak and strong parts. We propose that the different regimes can be understood in terms of differing causal mechanisms, which allows a broad distinction between correlations due to significant causes for two companies and incidental processes. It may appear too ambitious to seek to separate out correlations due to significant causes versus incidental processes. However, the current practice of relying on just price correlations from historic time series data does not take into account any possible causal mechanisms responsible for the observed behaviour; indeed such mechanisms may change with time, as markets are subject to structural change and different regimes may have been at work during the time period that is used to compile a database. Our aim is to show that substantial progress can be made with the above approach, utilizing technical tools from physics and the field of causal inference.

Specifically, in finance, we propose a definition of the *S*-values by partitioning the observed financial time series into different regimes. To this end, consider two securities *A* (for example, *Apple Inc.*) and *B* (for example, *Broadcom Inc.*) and a list of different *financial regimes* (to be explained shortly) with respect to a security such that, on a given day, one and only one financial regime prevails. The measurement outcomes are now generated by the simultaneous price changes in securities *A*, *B*. In particular, the outcome will be $a=1$ if security *A* has increased in price over a given time period and $b=1$ if security *B* has increased in price over the same time period. Similarly, decreases in price are denoted by $a=-1$ and $b=-1$, respectively.

For financial applications, the available history consists of public information and information that was possibly private initially and became public subsequently. Possible ways to determine financial regimes, by which the value of *x*, *y* is defined, would be by using an exogenous time series or the prices of the securities themselves. This allows partitioning the available data in a way that is analogous to the measurement settings in the standard Bell setup.

Restricting ourselves to financial price data only, an interesting choice of regimes is the distinction between *weak* and *strong* price change, whereby it is assumed that weak changes are due to incidental processes whereas strong changes are due to (ostensibly) shared, significant causal factors. Though not essential to the subsequent analysis, there are many ways to motivate these causal factors starting from known market mechanisms. For example, it is known that classical correlations tend to be higher during a market crash when investors may panic^28,38–42^. Under such circumstances, common causes driving correlations between many securities would be de-risking requirements and decreased collateral values.

To obtain a simple and symmetrical description, we separate large price changes from small changes by defining $x=1$ for each day in the time series when the price of security *A* has gone up or down by less than a fixed percentage $r_A$, and $x=0$ when the price of security *A* has changed by a larger amount. Days with $x=0$ are called strong days for security *A*. Similarly $y \in \{0,1\}$ is defined as a function of the price change in security *B*, over the same time period, using $r_B$ as threshold to separate weak from strong days. We think it is a reasonable intuition that strong price changes are due to significant events in the market, possibly unique to the pair of stocks considered, while weak changes are due to residual or incidental market processes. Note, analogous approaches can be envisaged in any domain of application, that is, we think that in the case of any variable we can (fairly generically) identify large vs. small changes, and so adopt definitions analogous to the ones just above - or exogenous variables could be recruited to separate out measurement regimes in the variables of interest.

With these definitions, the four *S*-values can be computed from Eqs. (1)–(4). Importantly, it is possible to derive variants of the Inequalities (5), for particular causal models, as shown below. If the empirical data shows violations of these inequalities, such causal models can be excluded in line with the leitmotiv of the field of causal inference^15–17^. Note, in the physics literature, a discussion of Bell inequalities is usually accompanied by careful consideration of whether an observed violation of the inequalities is due to ’genuine’ contextuality, versus signalling or direct influence (e.g.^43^). However, for the present purposes this distinction is irrelevant, because we aim at a general statistical technique capable of indicating a violation of certain causal mechanisms as described below.

The quantities $S_i$ are defined as linear combinations of four conditional expectations, which can be interpreted as correlations between the outcomes *a*, *b* under different regimes *x*, *y*. Out of these four *S*-values the $S_1$-value is the most interesting for us, because $S_1$ can be interpreted as correlation when strong change in at least one part of the system occurs. This can be seen directly from Eq. (1), as all correlations with at least one strong change (i.e., the regimes $xy = 00, \, 01$ and 10) are added, while the contribution with weak change in both parts of the system (i.e. $xy = 11$) is subtracted. So, $S_1$-values can be interpreted as a type of correlation (in the above specific sense), but where the contributions involving strong change on at least one part are separated from the contribution involving only weak parts.

We consider two ways to utilize *S*-values towards understanding the correlation between the variables of interest, here security prices. In both cases, assumptions about the correlation structure can be tested by comparing empirically measured *S*-values against theoretical *S*-values, derived on the basis of specific model assumptions. First, because the *S*-value can always be empirically computed independently of a parametric distribution model, we can examine very general causal models characterizing the interdependence between two securities, provided we can make a meaningful distinction between strong and weak change. Here we can derive specific bounds on possible *S*-values, which can be used to eliminate certain classes of models. In an application to finance, as we will see, the bound of 2 may be broken by dependencies between two stocks, but other bounds are implied by certain generative models like the Factor Models^44,45^ and the bivariate Gaussian model^46,47^. In both cases, models can be given a specific parametric form.

Can we apply our framework to acquire additional insights into these models or develop simplified tests of their applicability? Regarding Factor Models, we show that *S*-values computed conditionally on precisely known values of all contributing factors may not exceed 2. As this result holds for any arbitrary functional relationship between stock price returns and the contributing factors, one important and surprising message is this: in cases where a linear factor model is invalidated by finding conditional *S*-values above 2, then assuming a more complicated functional relationship for stock price change based on the same contributing factors will also be invalidated. Regarding the bivariate Gaussian distribution model, we show how *S*-values can be computed explicitly and how such values can exceed the classical limit of 2. Estimating *S*-values, as a function of classical correlation, the empirically observed *S*-value can be employed as a test of the adequacy of the Gaussian model. Overall, our approach brings together generative models of association with assumptions about the causal structure, allowing tests for both, in a seamless framework.

### Bell tests for generic causal models

A Bell analysis is underwritten by a causal network, encoding the assumptions of putative sources of influence between the two components of a system. In physics, with two binary questions for systems composed of two parts, it is straightforward to provide a corresponding causal network, consistent with the key assumptions of locality and free choice. In the finance case, more care is needed since the causal network has to additionally reflect the way assumptions about measurement regimes *x*, *y* interact with measurement outcomes *a*, *b*.

**Figure 1:**
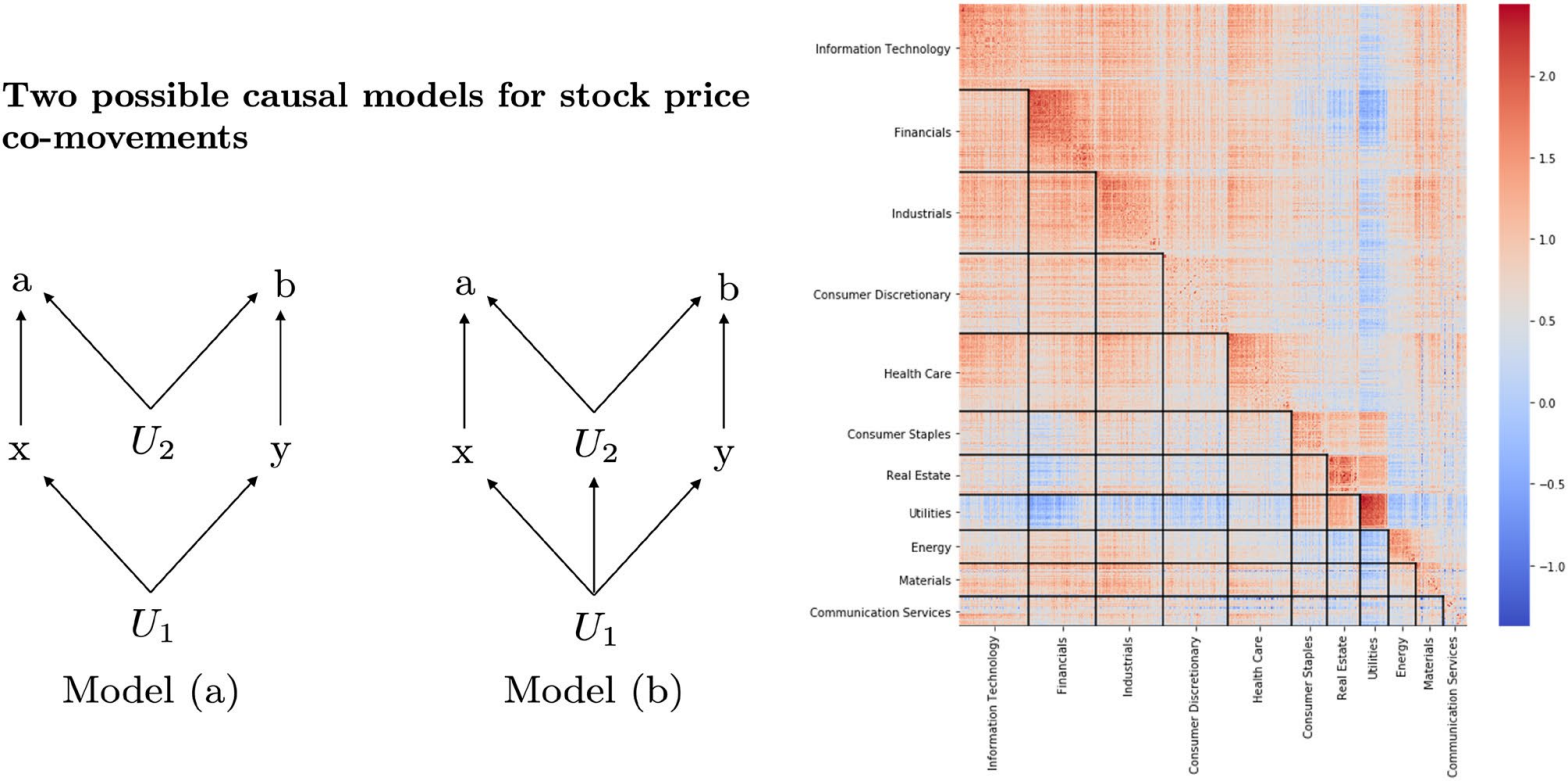
Two competing causal models. On the left-hand side two possible causal models for stock price co-movements are shown. Model (a) was inspired by the Bell experiments in quantum mechanics, while Model (b) is an extension of Model (a) with just one additional causal arrow. Both models may be proposed to describe stock price behavior in the regime of weak versus strong price changes. The right hand side shows a heatmap of $S_1$-values from daily closing price changes of S&P-500 stocks for the time period 4.5.2016 to 3.5.2019 grouped into 11 sectors according to the Global Industry Classification Standard (GICS) and ordered by descending strength of classical correlation within each sector. An identical threshold of $r_A = r_B = 1\%$ was used for each pair of stocks. The deep red indicates $S_1$-values above two. Those values falsify Model (a).

When the measurement regimes are categorized into a strong versus a weak price change, a natural choice for a simple causal model would be to postulate one unknown cause $U_1$ for driving the magnitude of price change (i.e. *x*, *y*) and a different unknown cause $U_2$ for driving direction (i.e. *a*, *b*). $U_1$ could be interpreted as a market volatility factor reflecting general uncertainty, while $U_2$ could be regarded as a measure of optimistic versus pessimistic market responses to new information. However, there is not a unique way in which even these two simple ideas can be translated into a causal model and Fig. 1 shows two variants of hypothetical causal mechanisms. Herein, $U_1$ is responsible for determining whether a trading day is strong, with a large price change in either direction. Cause $U_1$ is thus responsible for separating strong trading days from weak trading days (the latter reflecting incidental correlation) and thus influences only *x*, *y* directly. The second cause $U_2$ is responsible for determining price direction, i.e. whether stocks go up or down and therefore has direct influence on *a*, *b*.

How could we decide between Model (a) versus Model (b)? One may suspect that $U_1$ also has an effect on price direction, in which case in Fig. 1 an arrow from $U_1$ to $U_2$ is needed and hence Model (b) is more appropriate. However, one may also argue that Model (a) is simpler and should thus be preferred. Interestingly, it is possible to test via the *S*-values whether the simpler model provides a feasible option, because the simple Model (a) mathematically implies a Bell bound of 2, as stated in the following proposition (see Supplementary information for the proof).

#### Proposition 1

For the causal Model (a) in Fig. 1 the inequalities $|S_{\scriptscriptstyle i}|\le 2$ have to hold for all $i=1,\ldots \,,4$.

However, empirical values of $S_1$ can be substantially above 2, so that Model (a) is readily falsified as shown by the heatmap on the right-hand side of Fig. 1. The heatmap of $S_1$-values also illustrates the usefulness of the $S_1$-value to show the sectorial structure of the stock market. Below the diagonal, black dividing lines were added to show the industry sectors using the companies’ fundamental main operating business model.

In our application to the S &P 500, data for the $S_1$-value immediately falsified Model (a). In terms of examining different causal models, generally each quantity $S_{\scriptscriptstyle 1}, S_{\scriptscriptstyle 2}, S_{\scriptscriptstyle 3}, S_{\scriptscriptstyle 4}$ provides a testable opportunity to falsify a specific causal model, but a violation of the bound of 2 can occur at most in one of the four quantities:

#### Proposition 2

For a given statistic $\{ P( ab\, |xy)\}_{\scriptscriptstyle xy}$ not more than one of the four Inequalities (5) can be violated.

See Supplementary information for the proof. Regarding causal Model (a) in Fig. 1 the violation $|S_{\scriptscriptstyle 1}|>2$ therefore implies $|S_{\scriptscriptstyle i}|\le 2$ for $i=2,3,4$.

Other, more complex causal mechanisms than Model (a), are not ruled out by the data. For example, a causal connection from $U_1$ to $U_2$ may be assumed, leading to Model (b) in Fig. 1. This could be motivated in the financial area by considering that, for example, nervous and volatile markets may tend to interpret ambiguous news for stocks *A*, *B* in a pessimistic way, leading to a decline of stock prices. Formally, this leads to a link between causes for the magnitude of change (i.e. $U_1$) and causes for the direction of change (i.e. $U_2$ as it determines *a*, *b* and hence whether a price change is positive or negative). Despite the fact that Model (b) has just one causal arrow more than Model (a), Model (b) is fully general in the sense that, without further restrictions, any possible statistic for the four values *a*, *b*, *x*, *y* can be generated from it, as the following proposition shows.

#### Proposition 3

Let ${\tilde{P}}$ be an arbitrary joint probability distribution of the quadruplets (*a*, *b*, *x*, *y*) and let *P* denote the joint probability distribution generated by Model (b) for those quadruplets. Then, Model (b) can be specified in a manner that yields $P = {\tilde{P}}$. This can be achieved by defining $U_1$ appropriately and setting $U_2 := U_1$, so one general cause suffices to generate any arbitrary distribution ${\tilde{P}}$.

The proof of Proposition 3 is given in Supplementary information. It shows that Model (b) achieves its generality essentially by having a causal connection from one unkown cause to all observed values *a*, *b*, *x*, *y* and by allowing arbitrary probability distributions for the unkown cause. So, Model (b), in its general form, provides a generic class of models. Because of this generality, it cannot be applied directly. In the next section, we will therefore explore three pertinent special cases to restrict Model (b).

## Bell tests for specific parametric causal models

So far we have not identified a causal model, which lends itself to an applicable description of the empirical data and/or application of more specific generative models. We want to achieve this by exploring specific parametric special cases of the generic Model (b). First, we start with a situation where the unknown causes have a very simple parametric expression through dichotomous or uniformly distributed values. Second, as another special case of Model (b), we consider Factor Models, i.e. descriptions of stock price returns through a linear combination of different observable factors. Third, we examine the bivariate Gaussian distribution model as a generative model, in which stock price change is driven by a fundamental drift and a random overlay of volatility modelled by Brownian Motion.

1. A generating model with dichotomous causes. As outlined, we first consider the implications from allowing only simple parametric distributions for the causes in Model (b). As shown in the first part of the following Proposition 4, any distribution of quadruplets can be generated by a special case of Model (b), where we use only one dichotomous unknown cause and one uniformly distributed common error term driving the behavior of *a* and *b*. In particular, a causal graph equivalent to Model (b) can be specified, where the causal arrows concerning weak vs. strong days (parameters *x*, *y* expressing for example volatility) can be separated from the causal arrows for upward vs. downward price change (parameters *a*, *b* expressing for example market direction), by the simple addition of a uniformly distributed common error term.

With Model (b) it is also easy to generate the full algebraically possible range of $S_1$-values without being restricted by a bound, like the Tsirelson bound in quantum mechanics. In fact, two separate dichotomous causes specified by only one parameter are sufficient to achieve this, as shown in the second part of Proposition 4. We give an explicit corresponding parametrization in the proof of Proposition 4 in Supplementary information, which is amenable to further generalisations, and outlines a way by which Model (b) can be made practically useful as a parametric model for an observed statistic.

### Proposition 4

Assuming bivalued unknown causes in Model (b) in Fig. 1, the following holds true:

1. For any arbitrary joint probability distribution ${\tilde{P}}$ of the quadruplets (*a*, *b*, *x*, *y*), identical bivalued causes $U_1$ and $U_2$ can be defined such that the joint probability distribution generated by Model (b) equals ${\tilde{P}}$ up to an independent, uniformly distributed error term common to *a* and *b*.
2. An explicit parametrization for two separate causes $U_1$ and $U_2$ with only one free parameter can be given such that the $S_1$-value computed from Model (b) can attain every number in the interval $[-4,+4]$.

To prove the second part of Proposition 4, we proceed by directly parameterizing the strength of the causal links in Model (b). Specifically, different parameters concern the strength of the links representing significant causes versus residual processes. Our approach allows us to compute $S_1$ as a product $S_1 = 4(1-2\gamma )(1-2\epsilon )$. See the proof of Proposition 4 in Appendix A of the Supplementary information for a definition of the parameters $\gamma$ and $\epsilon$. Intuitively, parameter $\gamma$ can be thought of as quantifing the strength of the link between the common causes $U_1$ and $U_2$ in Model (b) in Fig. 1, while parameter $\epsilon$ corresponds to the pattern of outcomes, i.e. the links between $U_2$ and *a*, *b* in that causal model.

The model from the proof of Proposition 4 should be seen as an illustrative example. It is a compromise between a small number of parameters and sufficient flexibility. In this simple model, possible $S_1$-values span the entire algebraic range $[-4,+4]$, while the other quantities $S_2, S_3, S_4$ vanish, but it can be extended to situations with non-zero $S_2$-, $S_3$- and $S_4$-values by introducing additional free parameters. Despite its simplicity, the specified model allows us to generate all theoretically possible $S_1$-values, if the parameters $\gamma$ and $\epsilon$ are unrestricted. Note, particular empirical domains of application might allow us to specialize the model to reduced ranges for $\epsilon , \gamma$, thereby restricting the possible range for $S_1$ as well. In contrast to the prediction from quantum mechanics^7^, this approach generates no general Tsirelson bound for $S_1$.

2. Factor Models. Factor Models aim to explain the returns of single stocks by one or more observable common factors. Such common factors may simply be the return of a broad market index, like the S &P 500, or more complex factors, such as the return of a diversified portfolio of small stocks minus the return of a diversified portfolio of large stocks, see^44,45^. Formally, a linear Factor Model assumes that the returns of two stocks $R^A, R^B$ are given by the relationships
  6 $$\begin{aligned} R^A= & {} \alpha ^A + \sum _{j=1}^m \beta ^A_j F_j + e^A, \end{aligned}$$
  7 $$\begin{aligned} R^B= & {} \alpha ^B + \sum _{j=1}^m \beta ^B_j F_j + e^B. \end{aligned}$$
  Here, $F_1,\ldots ,F_m$ are common factors that are observable on any trading day, whereas $\alpha ^A , \alpha ^B$ and $\beta ^A_1,\ldots , \beta ^A_m, \beta ^B_1,\ldots , \beta ^B_m$ are constants. Differences (residuals) between the observed stock returns $R^A, R^B$ and the linear predictions are denoted by $e^A, e^B$ and are assumed to be independent.

If the assumed linear relationship is unrealistic, the Factor Model can be generalized to

8 $$\begin{aligned} R^A= & {} f_A \left( F_1, \ldots , F_m, e^A \right) , \end{aligned}$$

9 $$\begin{aligned} R^B= & {} f_B \left( F_1, \ldots , F_m, e^B \right) \end{aligned}$$

by using two arbitrary functions $f_A$, $f_B$.

**Figure 2:**
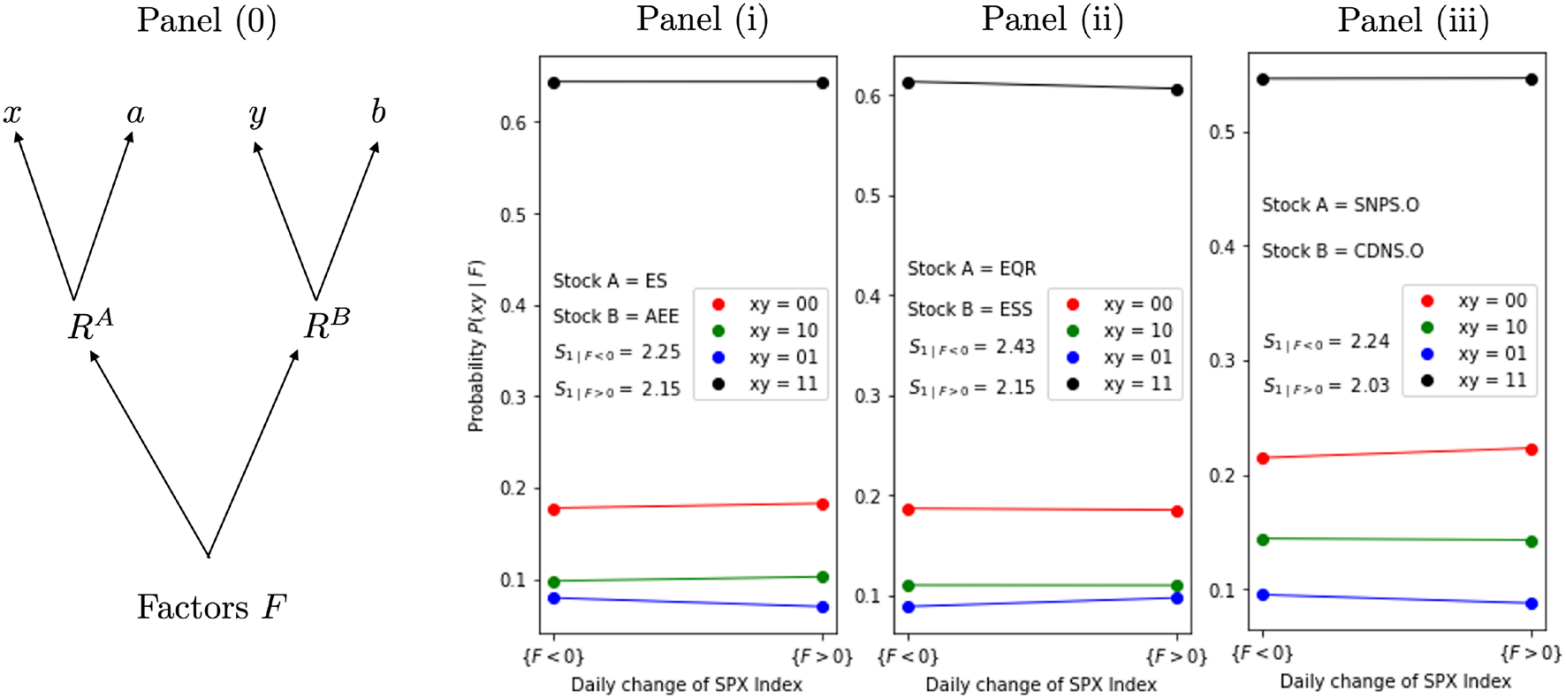
Factor Models. Panel (**0**) illustrates the Factor Model described in Eqs. (8) and (9), where the factors *F* generate the stock price returns $R^A$ and $R^B$ from which *x*, *a* and *y*, *b* are derived. Residuals are not shown in the diagram and appear as noise terms in the equations. Panels (**i**), (**ii**) and (**iii**) illustrate empirical tests of the factor model. These panels are based on the daily price change of the general S&P-500 market index as the explaining factor for market price change during the time period 4.5.2016 to 3.5.2019. Each of the three panels shows the conditional probabilities $P(xy | \cdot )$ for the four regimes $xy = 00, 10, 01, 11$ for a different pair of stocks. Again, a threshold of $1\%$ is used to distinguish days with weak price change from days with strong price change. We compare the value of $P(xy | F<0)$, i.e. the probability on days with negative returns in the S&P-500, against $P(xy | F>0)$, i.e. the probability on days with positive returns in the S&P-500. Horizontal lines in the panels show equal probabilities. Panel (**i**) shows the stock pair *ES (Eversource Energy)* and *AEE (Ameren Corp)* with conditional $S_1$-values of $S_{1 | F<0 } = 2.25$ and $S_{1 | F>0 } = 2.15$. Panel (**ii**) shows the same analyses for the pair of stocks *EQR (Equity Residential)* and *ESS (Essex Property Trust)*, while the righthand Panel (**iii**) shows the analyses for the pair of stocks *SNPS.O (Synopsys Inc.)* and *CDNS.O (Cadence Design Systems Inc.)*.

As Model (b) can generate any distribution of quadruplets, Factor Models can be seen as a special case of Model (b) for the purpose of analyzing *S*-values. However, Factor Models provide a more specific causal story, because Factor Models connect *x* and *a*, as well as *y* and *b* in a special way, as the former pair (*x*, *a*) is derived from $R^A$ only, while the latter pair (*y*, *b*) is solely generated from $R^B$, as illustrated in the causal diagram in Fig. 2. Interestingly, Factor Models allow a Bell bound conditional on the common factors, as the following proposition shows.

### Proposition 5

With given arbitrary functions $f_A, f_B$, random variables $F_1,\ldots ,F_m$ (“factors“) and random variables $e^A, \, e^B$ (“residuals“), Eqs. (8) and (9) define two stock return processes $R^A, \,R^B$, from which the variables *a*, *b*, *x*, *y* can be computed.

Let us denote the four *S*-values computed conditionally on the values of the factors as $S_{i|F}$. If the residuals are stochastically independent, then we obtain

$$\begin{aligned} |S_{\scriptscriptstyle i | F}|\le 2&\qquad \text {for}\ \ i=1,\ldots \,,4. \end{aligned}$$

See Supplementary information for the proof. Importantly, the derivation of the Bell bound for the conditional *S*-values holds even in the case of a non-linear Factor Model and is independent of the number of factors used.

The Bell bound in Proposition 5 only holds conditionally on all factor values $F = (F_1,\ldots , F_m)$. An interesting conundrum arises: We have already seen that for many pairs of stocks the value of $S_1$ as computed from the data exceeds the value of 2. So, how is it possible that when computing $S_1$-values conditionalised on the factors *F*, all these conditional $S_1$-values may not exceed 2? One possible answer is that the specific Factor Model is incorrect in most cases. However, there is another, subtler possibility. In fact, this analysis reveals instances of Simpson’s paradox. As we show with an illustrative example in Appendix B, it is possible to have $S_{1|F} \le 2$ conditional on different factor values, but when computing $S_1$ for all data, $S_1>2$, because

10 $$\begin{aligned} S_i \ne \sum _{F} S_{\scriptscriptstyle i | F} P(F) \end{aligned}$$

may hold.

Proposition 5 is a powerful result concerning the implications of applying a Factor Model to a particular pair of stock prices. It links Factor Models with our framework, via the conditionalisation of the *S*-values on specific values of *F*. Assume that we want to construct a specific Factor Model for two stocks and a single observable factor *F*, such as a general market index like the S&P 500. Then, if there exists an instance for the variable *F* in which $S_{1|F} > 2$ holds, we have to conclude that the considered Factor Model is invalid. Current practice concerning Factor Models typically involves linear regression (and so an assumption of linear relationship between price indices and the single stocks), but Proposition 5 is not restricted in this way: Proposition 5 encompasses any functional relationship between the price indices and the additional variables *F*, so that $S_{1|F}>2$ indicates that something is missing in *any* function linking single stock price returns to a particular factor *F* (in that the residuals from the corresponding Eqs. (8) and (9) would not be independent). This holds also true if more than one factor is used. An important implication is that if one does not have the right factors when setting up a linear model, then moving to non-linear models with complicated functional relationships would generally not help.

The values in Eqs. (8) and (9) have a continuous distribution, so that conditioning on specific single point values is not practical. To apply Proposition 5 with real data, the first step is to select appropriate intervals for the factors *F*, such that the measurement settings are stable across intervals, i.e. $P(xy|F) = P(x,y|F) = P(x,y)$. With a stable probability distribution for the measurement settings, i.e. constant *P*(*xy*|*F*) on a set of factor values, Proposition 5 holds, as shown in Appendix C of the Supplementary information. In practice, there would be a trade-off between choosing small intervals, yielding constant probabilities versus intervals that contain a reasonably large number of data points.

For the present examples, we used three different pairs of stocks and divided the range of value changes in the single factor S&P 500 into two intervals, distinguishing between days with positive returns of the S&P 500 and days with negative returns, i.e. we look at $P(xy|F>0)$ versus $P(xy|F<0)$. It can be seen, from the almost horizontal lines in Fig. 2, that the assumption of measurement setting independence is approximately valid. For different pairs of stocks we observe conditional $S_1$-values above 2 both on the positive and the negative interval. This indicates a failure of the specific Factor Model for the observed price change of the two stocks, when assuming a coarse graining of the S&P 500 index by just distinguishing between positive and negative returns. Therefore, it is not possible to have a complete explanation of the observed (Bell) statistics of the two stocks under consideration, with this specific Factor Model, based on a two-interval coarsening of S &P 500. That is, a Factor Model for these two stocks based on just whether S &P 500 was up or down on different trading days fails.

**Figure 3:**
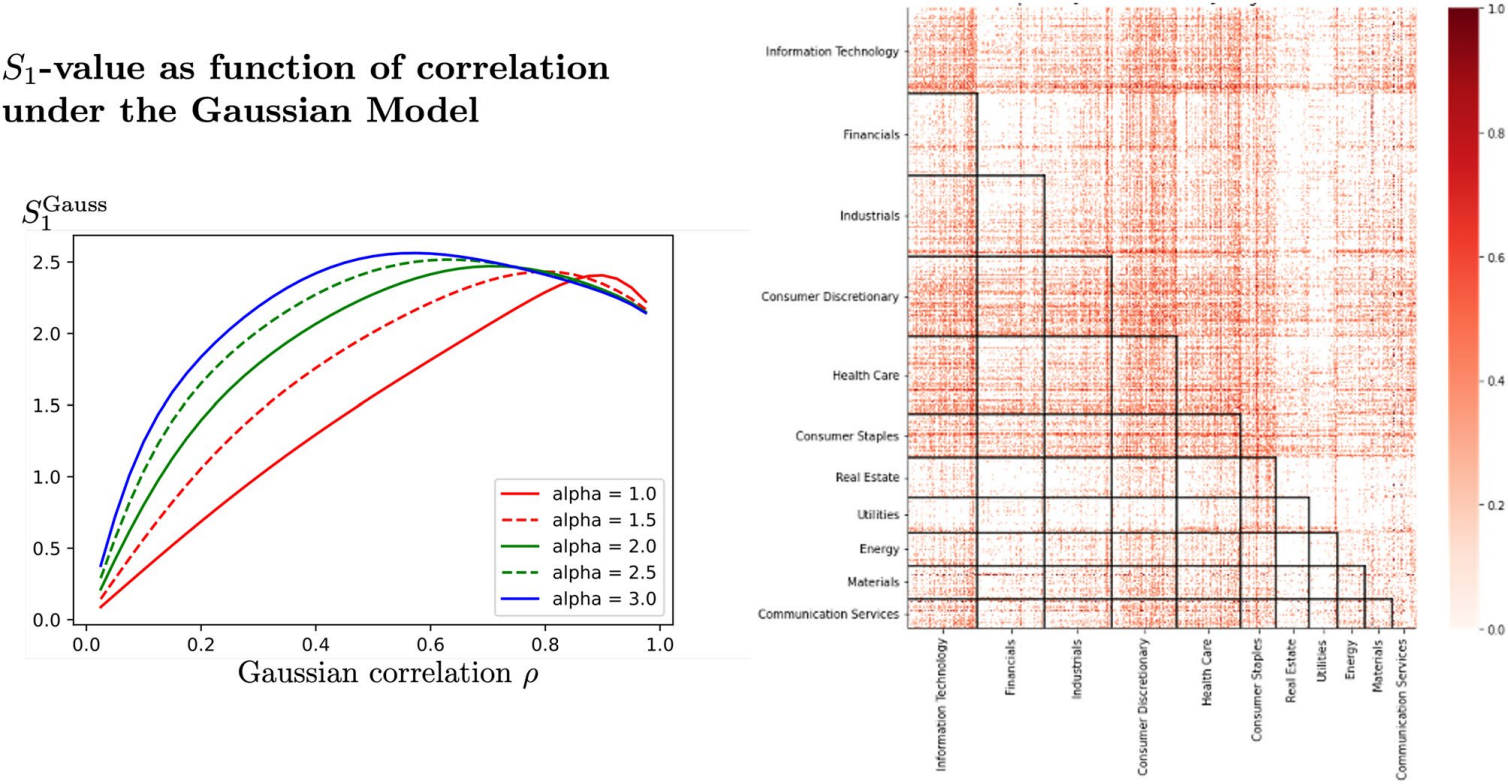
Gaussian Models. The lefthand side shows the $S_1^\text {Gauss}$-values computed via Monte Carlo simulation as functions of Gaussian correlation $\rho$, with thresholds taken as alpha multiplied by the respective volatility (i.e. $r_A = \alpha \sigma _A$ and $r_B = \alpha \sigma _B$, with $\alpha$ chosen in the range from 1 to 3). The righthand side shows a heatmap of the positive excess $\Delta = S_1^\text {emp} - S_1^\text {Gauss}$ from daily closing price changes of S&P-500 stocks using the same time period, GICS sector classification and ordering as in Fig. 1. For each pair of stocks, $\Delta$ was computed by setting the threshold, that separates strong from weak days, to the stock’s daily volatility, as it was observed over the entire time period. Equation (1) was used to compute $S_1^\text {emp}$ from the time series of historic stock prices, while the $S_1^\text {Gauss}$-value used was based on Monte Carlo simulations (as illustrated in Fig. 3 for positive values), with $\rho$ set to the historic correlation between the daily returns of the two stocks under consideration.

In general, if the condition of measurement setting independence is fulfilled, i.e. constant conditional probabilities $P(xy | \cdot )$ occur across different ranges of factor values (see Proposition 7 in Appendix C of the Supplementary information), the degree by which $S_{1}$ exceeds 2 can be seen as a measure of the non-applicability of the Factor Model. While the above examples in Fig. 2 use a very rough coarse graining of the Factor, by partitioning the range of S&P 500 index values into just two intervals, a finer partitioning is of course possible and could yield a stronger conclusion. However, fulfilling the condition of constant conditional probabilities $P(xy | \cdot )$ becomes harder, when a fine partition with many sub-intervals is used. An important direction for future work is extending Proposition 7 to situations when the probabilities $P(xy | \cdot )$ vary across the chosen intervals. Also, note that such analyses can be easily extended by considering other variables for a Factor Model, e.g. as in multi Factor Models like the 3- or the 5-Factor Model by Fama and French^44,45^.

3. Gaussian Models. The present approach can be utilized to examine the validity of different generative models for associations between securities. Apart from Factor Models, a particularly influential one is the bivariate Gaussian model, which is frequently used in continuous time financial theory for pricing and hedging of derivative securities and for optimal consumption over time, see^48–51^.

The Gaussian model can be expressed for two stocks as

11 $$\begin{aligned} dS^A(t)= & {} S^A(t) (\sigma _A dW^A(t) + \mu _A dt), \end{aligned}$$

12 $$\begin{aligned} dS^B(t)= & {} S^B(t) (\sigma _B dW^B(t) + \mu _B dt), \end{aligned}$$

with two correlated Brownian Motions $W^A, W^B$, two positive numbers $\sigma _A, \sigma _B$ as price volatilities, and $\mu _A, \mu _B$ as price drifts. With the simplification of assuming zero risk free interest rates and zero price drift, three model parameters remain, $\sigma _A, \sigma _B$ and the correlation $\rho$ between the two Brownian Motions.

The density function, for the joint distribution of the logarithmic returns of the two stocks over a short time interval

$$\begin{aligned} R^A = \ln \left( \frac{S^A(t)}{S^A(t-1)} \right) , \, R^B = \ln \left( \frac{S^B(t)}{S^B(t-1)} \right) , \end{aligned}$$

is given by

$$\begin{aligned} p_\rho (v, w) = \frac{ \exp \left( - \frac{1}{2(1-\rho ^2)} \left( \frac{v^2}{\sigma _A^2} +\frac{w^2}{\sigma _B^2} - 2\frac{\rho v w}{\sigma _A \sigma _B} \right) \right) }{2\pi \sigma _A \sigma _B \sqrt{1-\rho ^2}}. \end{aligned}$$

From this density function, the four expectation values that make up the *S*-value in Eqs. (1)–(4), can be computed, such as, for example:

$$\begin{aligned} \langle a b \rangle _{00}= & {} {\mathbb {E}} \left[ \text {sign} ( R^A ) \text {sign} ( R^B ) 1_{ | R^A |> r_A } 1_{ | R^B | > r_B } \right] . \end{aligned}$$

If the thresholds $r_A$ and $r_B$ are taken as constant multiples of the volatilities $\sigma _A$ and $\sigma _B$, fomulae for the *S*-values are possible that contain only the Gaussian correlation $\rho$ as a free parameter, see Supplementary information.

### Proposition 6

If the price of two securities follows the bivariate Gaussian model in Eqs. (11)–(12) and if the thresholds that separate weak from strong days are given as $r_A = \alpha \sigma _A$ and $r_B = \beta \sigma _B$ with positive constants $\alpha , \beta$, then the *S*-values can be computed via analytic expressions. It holds for example that

$$\begin{aligned} \langle a b \rangle _{00}= & {} \frac{ \int _{\gamma _x}^\infty \int _{\gamma _y}^\infty e^{-v^2 - w^2} \sinh (2\rho v w) dv dw}{\int _{\gamma _x}^\infty \int _{\gamma _y}^\infty e^{-v^2 - w^2} \cosh (2\rho v w) dv dw } \\= & {} \frac{ \sum _{n=0}^\infty c_{2n+1} \Gamma (n+1, \gamma _x^2 )\Gamma (n+1, \gamma _y^2 ) }{ \sum _{n=0}^\infty c_{2n} \Gamma (n+1/2, \gamma _x^2 ) \Gamma (n+1/2, \gamma _y^2 )} \end{aligned}$$

where $\Gamma (s, x) = \int _x^\infty t^{s-1}e^{-t} dt$ denotes the incomplete gamma function and

$$\begin{aligned} \gamma _x = \frac{\alpha }{ \sqrt{2(1-\rho ^2)} },\; \gamma _y = \frac{ \beta }{ \sqrt{2(1-\rho ^2)} }, \; c_{k} = \frac{2^k \rho ^{k}}{k!}. \end{aligned}$$

The other expectation values $\langle a b \rangle _{10}, \langle a b \rangle _{01}, \langle a b \rangle _{11}$ have analogous analytic expressions leading to an analytic formula for $S_i^\text {Gauss}$ for all $i=1,2,3,4$ in the Gaussian model, as shown in Supplementary information.

Figure 3 illustrates $S_1^\text {Gauss}$ as a function of Gaussian correlation $\rho$, if the same multiple of volatility is chosen for the thresholds of the two securities. The Gaussian model does allow strong levels of association between two securities and readily yields values $S_1^\text {Gauss}$ above 2, however, it does not exhaust the full range of possible $S_1$-values $[-4, 4]$. In fact, $S_1$-values that are possible under the Gaussian model lie substantially below 4 and may therefore not explain high empirical $S_1$-values. If the empirically determined value $S_1^{\text {emp}}$ is above the curves shown in Fig. 3, then we may conclude that the data would offer a refutation of this model. Figure 3 illustrates the excess of empirical $S_1$-values over $S_1^\text {Gauss}$ for S&P-500 stocks with red colors. While the amount of information in this figure is too much to make it readily applicable, it does illustrate that using the $S_1$-value offers a simple test of the applicability of the Gaussian model, for any two stocks.

## Conclusion and discussion

A key objective in most scientific domains is to understand the causal structures which give rise to observed correlations. For example, in finance, what are the factors that drive risk and return between the components of financial portfolios? Finding asset allocations and risk diversification strategies that lead to a smooth and balanced outcome under different financial regimes is key for financial stability and economic prosperity. Studying the association and mechanisms between joint price changes in financial instruments is therefore of high importance.

Our approach has been to explore an established framework from physics for linking assumptions about causal structure to correlations. There is a long history of cross-fertilization between physics and other disciplines. Regarding our chosen example in finance, a notable application concerns the heat equation to the pricing and hedging of financial derivatives^48^. Quantum methods have also been applied to problems in social science, cognitive modelling, games and finance^20,52–57^.

Regarding correlations, Bell’s approach is the most influential framework for understanding the way particular causal models can be linked to observed correlations in nature. It is a framework and a general method for understanding the structure in the correlation between two components of a system, afforded by an underlying causal model^58–60^. However, its formulation does not depend on the laws of quantum mechanics and is in principle open to applications in any domain (concerning economics and finance, see, for example,^15–17,61–63^).

The main difference between the application of Bell’s framework in physics and applications in other areas is that in the former case it is employed as a test of macrorealistic models, whereas in applications on the macroscopic domain, such as finance, realism is a given, so that violations of Bell bounds have to be interpreted in the terms of a putative underlying causal network. This yields a fairly generic approach recognizing that correlations between two variables can occur because of shared causes or because of random fluctuations, where only the former is typically of interest. In finance, the variables of interest would be the price of two securities, so that our approach essentially assumes a separation between correlations resulting from significant shared causes from correlations assumed to be due to residual market processes. Such a separation can be realized using Bell’s framework.

The partitioning of (price) data into different regimes allows different conclusions regarding the causal model of association in these regimes. This opens the route to explore and possibly refute competing causal models from the available data. We offered a basic example: when *x*, *y* are defined by the magnitude of price change, one of the two causal models in Fig. 1 can be readily excluded from observation on the basis of Proposition 1. The remaining causal model from Fig. 1 is very general, but can be given a parameterization to allow a simple description of observed $S_1$-values, as shown in Propositions 3 and  4. These methods are not specific to finance and can be easily generalized to any domain.

There are many alternative ways to restrict Model (b), including by utilizing domain-specific theory. In finance, two influential models are Factor Models and Gaussian Models, though note again that the applicability of these models is quite general (they are particular ways to model the association between variables). For Factor Models we have shown in Proposition 5 that conditional *S*-values are subject to the Bell bound, provided the residuals are stochastically independent. This provides interesting testing possibilities in empirical data sets in which the measurement settings *x*, *y* remain stable across chosen intervals of factor values. As the mathematical result does not require a linear relationship between factors and stock price returns and as it holds for any number of factors, Proposition 5 underlines the importance of choosing the right factors with independent residuals, when setting up a factor model. Proposition 5 thus offers potential for a very general test of proposals for Factor Models based on particular combinations of factors.

Regarding the widely used Gaussian model, Proposition 6 shows how the present framework can be employed so that limits on the $S_1$-value can be used to probe the Gaussian distribution assumption. While Gaussian models are known to underestimate the probability of extreme events, the $S_1$-value can be computed independently of the Gaussian assumption and, where empirical $S_1$-values outside the range attainable by Gaussian models are found, the inappropriateness of the Gaussian assumption follows. The present approach offers an alternative, simple way to test Gaussian models, which complements existing methods, such as, specifically for finance^64–66^, and can be generalised to variants of the Gaussian model approach.

The present paper aims to outline possible applications of Bell’s method as a general framework for linking causal assumptions to observed correlations. Even when it is desirable to restrict analyses to a single variable of primary interest, such as stock price, there is a rich range of possibilities one could employ, depending on the focus of interest. While in this paper only one regime was considered, the list of possible regimes can be readily extended. Following from our example in finance, to study, for example, causal mechanisms of market crashes, an asymmetric definition, where *x*, *y* are set to the value of zero only in the case of a large price decline could be used.

With the present advances in computational power and theoretical methods of machine learning, applications of data mining algorithms to finance are often attempted. However, while the amount of data may seem large, the available time periods are sometimes short and the relevant environments may not be static. For example, in finance, with ongoing changes in regulation, investor behavior, as well as fiscal and monetary policy, the available time series data generally results from different causal regimes. To find appropriate quantitative models for learning it may also be important to incorporate human knowledge regarding economic, political and market mechanisms into a machine learning approach.

Another consideration is that the present method can be generalised so that the regimes can be defined via additional variables, to take into account particular hypotheses for specifying the different measurement regimes. In finance, such variables might correspond to known economic, political, regulatory, and market mechanisms. The important point is that, in specifying the underlying generative causal model, there would be many cases where different models imply different restrictions on the ensuing correlations, that can be tested using the present approach. In particular, a Bell test may reveal when a certain formalisation of human knowledge is at odds with observed statistical facts. The language of Bayesian networks and the causal model approach may thus help to build better models, based on statistical data and assumptions about putative causal mechanisms. Ultimately, more informative measures of association may be derived, by simple choices concerning the underlying causal mechanisms for the variables of interest.

Overall, we have shown how Bell’s framework in physics can be adapted to offer a measure of association between variables in any domain, focused on a distinction between strong and weak changes. We explored various causal models formalising an intuition of changes due to shared, substantial causes vs. incidental processes and illustrated various ways in which an initial causal model proposal could be refined. Additionally, we considered two well-known generative models in our application domain, based on factors driving market returns or on a bivariate Gaussian distribution, to describe co-movements in security prices. In both cases, we showed how our framework can provide simple tests for the validity of the chosen model in different cases. Thus, the $S_1$-value extends the concept of correlation both instrumentally and in terms of the underlying supporting theory.

## Supplementary Information

Supplementary Information.

## Data Availability

The data that support the findings of this study are available from www.refinitiv.com but restrictions apply to the availability of these data, which were used under license for the current study, and so are not publicly available. Data are however available from the corresponding author upon reasonable request and with permission of *refinitiv*.
