# Author Correction: Bell correlations outside physics

**DOI:** 10.1038/s41598-023-32723-0

**Published:** 2023-04-04

**Authors:** C. Gallus, E. M. Pothos, P. Blasiak, J. M. Yearsley, B. W. Wojciechowski

**Affiliations:** 1 Technische Hochschule Mittelhessen, 35390 Gießen, Germany; 2 City, University of London, London, EC1V 0HB UK; 3 Institute for Quantum Studies, Chapman University, Orange, CA USA; 4 Institute of Nuclear Physics Polish Academy of Sciences, 31342 Kraków, Poland; 5 Institute of Applied Psychology, Jagiellonian University, 30348 Kraków, Poland

Correction to: *Scientific Reports* 10.1038/s41598-023-31441-x, published online 16 March 2023

In the original version of this Article, the author name P. Blasiak was inadvertently duplicated in the author list.

The original Article has been corrected.

