# Supplementary material for: Bell correlations outside physics
Source: Sci Rep. 2023 Mar 16;13:4394. doi: 10.1038/s41598-023-31441-x (PMC10020465; doi:10.1038/s41598-023-31441-x)
Supplement: Supplementary file 1 — Supplementary Information. [file 41598_2023_31441_MOESM1_ESM.pdf]

SUPPLEMENTARY INFORMATION

We first prove Propositions 1, 3, and 5, proofs for the other propositions can be found in Appendix A. Appendix B shows how Simpson's paradox can occur in the computation of the S_1-value. Appendix C extends Proposition 5 to intervals. Numbering of equations, figures and references follows the main text.

Proof of Proposition 1

With the dependencies shown in the causal model (a) in Fig. 1 it holds that

$$\begin{aligned} P(ab\,|xyU_1U_2) &= P(ab\,|xyU_2) = P(a\,|xU_2)P(b\,|yU_2), \\ P(U_1U_2\,|xy) &= P(U_1\,|xy)P(U_2). \end{aligned}$$

This follows formally from the d-separation rules applied to the causal Model (a) shown in Fig. 1, see [13]. Therefore the expectation value $\langle ab\rangle_{xy}$ computes as

$$\begin{aligned} \langle ab\rangle_{xy} &= \sum_{a,b} ab\, P(ab\,|\,xy) \\ &= \sum_{a,b}\sum_{U_1,U_2} ab\, P(ab\,|\,xyU_1U_2)P(U_1U_2\,|xy) \\ &= \sum_{a,b}\sum_{U_1,U_2} ab\, P(a\,|xU_2)P(b\,|yU_2)P(U_1\,|xy)P(U_2) \\ &= \sum_{U_2}\sum_{a,b} ab\, P(a\,|xU_2)P(b\,|yU_2)P(U_2)\sum_{U_1} P(U_1\,|xy) \\ &= \sum_{U_2}\left(\sum_{a} a\, P(a\,|xU_2)\right)\left(\sum_{b} bP(b\,|yU_2)\right)P(U_2) \\ &= \sum_{U_2} \langle a\rangle_{xU_2}\langle b\rangle_{yU_2}P(U_2)\,, \end{aligned}$$

where in the last line we have used the definitions

$$\begin{aligned} \langle a\rangle_{xU_2} &= \sum_{a} aP(a\,|xU_2) \in [-1;+1]\,, \\ \langle b\rangle_{yU_2} &= \sum_{b} b\, P(b\,|yU_2) \in [-1;+1]\,. \end{aligned}$$

Thus,

$$\begin{aligned} |S_1| &= |\langle ab\rangle_{00} + \langle ab\rangle_{01} + \langle ab\rangle_{10} - \langle ab\rangle_{11}| \\ &\leq \sum_{U_2} |\langle a\rangle_{0U_2}\langle b\rangle_{0U_2} + \langle a\rangle_{0U_2}\langle b\rangle_{1U_2} + \langle a\rangle_{1U_2}\langle b\rangle_{0U_2} \\ &\qquad -\langle a\rangle_{1U_2}\langle b\rangle_{1U_2}|\, P(U_2) \leq \sum_{U_2} 2\,P(U_2) = 2, \end{aligned}$$

where we have used the standard algebraic bound on the expression $|a_0b_0 + a_0b_1 + a_1b_0 - a_1b_1| = |a_0(b_0+b_1) + a_1(b_0 - b_1)| \leq 2$, which holds whenever there are four real numbers a_0, a_1, b_0, b_1 in the intervall $[-1, 1]$. The proof of $|S_i| \leq 2$ for $i = 2, 3, 4$ is similar. $\square$

Proof of Proposition 3

As the four values a, b, x, y are all dichotomous, there are 16 possible quadruplets (a, b, x, y). An arbitrary joint probability distribution $\tilde{P}$ on the set of all possible quadruplets is therefore fully defined by 16 non-negative numbers, such that the following normalisation condition holds:

$$\sum_{\tilde{a},\tilde{b}\in\{\pm1\}}\ \sum_{\tilde{x},\tilde{y}\in\{0,1\}} \tilde{P}(a=\tilde{a}, b=\tilde{b}, x=\tilde{x}, y=\tilde{y}) = 1$$

The proposition is proven if we can define a discrete Kolmogorov probability space (Ω, P) together with six variables U_1, U_2, x, y, a, b such that $P = \tilde{P}$ holds and such that the definition of the variables is consistent with the arrows shown for Model (b) in Fig. 1.

Such a space can simply be defined by using the set of the 16 possible quadruplet values as $\Omega := \{\pm 1\} \times \{\pm 1\} \times \{0,1\} \times \{0,1\}$, and by defining the probability as

$$P(\{\omega\}) := \tilde{P}(\tilde{a}, \tilde{b}, \tilde{x}, \tilde{y}) \text{ for } \omega = (\tilde{a}, \tilde{b}, \tilde{x}, \tilde{y}) \in \Omega.$$

This definition gives $P = \tilde{P}$.

The two unknown causes in Model (b) are simply taken as the identity map $\Omega \to \Omega$, written formally as $U_1(\omega) := \omega$ and $U_2(U_1(\omega)) := U_1(\omega) = \omega$. The random variables x, y, a, b are defined as projections

$$x(\omega) := \tilde{x},\ y(\omega) := \tilde{y},\ a(\omega) := \tilde{a},\ b(\omega) := \tilde{b}$$

for all $\omega = (\tilde{a}, \tilde{b}, \tilde{x}, \tilde{y}) \in \Omega$. As these definitions can also be written as

$$x(U_1) = \tilde{x},\ y(U_1) = \tilde{y},\ a(U_2, x) = \tilde{a},\ b(U_2, y) = \tilde{b}$$

they are consistent with the arrows shown for Model (b) in Fig. 1. □

Proof of Proposition 5

We assume a functional model for the two stock returns R^A, R^B that is given by Equations (8) and (9), namely

$$R^A = f_A\left(F_1, ..., F_m, e^A\right),$$
$$R^B = f_B\left(F_1, ..., F_m, e^B\right).$$

with two arbitrary functions f_A, f_B depending on the common factors $F_1, ..., F_m$. The key assumptions is that the residuals e^A, e^B are stochastically independent. From R^A, R^B the random variables a, b, x, y are computed as usual as

$$x := 1_{|R^A| \le r_A},\ y := 1_{|R^B| \le r_B},$$
$$a := \text{sign}(R^A) \text{ and } b := \text{sign}(R^B).$$

We have to prove that the inequalities

$$-2 \le S_{i|F} \le 2 \tag{13}$$

hold for all $i = 1, \ldots, 4$, where $F := (F_1, ..., F_m)$ and where $S_{i|F}$ denotes the S_i-value conditional on all factor values $F_1, ..., F_m$.

Formally, the values $S_{i|F}$ are defined as a conditional version of Equations (1) to (4) by

$$\begin{aligned}
S_{1|F} &= \langle ab\rangle_{00|F} + \langle ab\rangle_{01|F} + \langle ab\rangle_{10|F} - \langle ab\rangle_{11|F}\,,\\
S_{2|F} &= \langle ab\rangle_{00|F} + \langle ab\rangle_{01|F} - \langle ab\rangle_{10|F} + \langle ab\rangle_{11|F}\,,\\
S_{3|F} &= \langle ab\rangle_{00|F} - \langle ab\rangle_{01|F} + \langle ab\rangle_{10|F} + \langle ab\rangle_{11|F}\,,\\
S_{4|F} &= -\langle ab\rangle_{00|F} + \langle ab\rangle_{01|F} + \langle ab\rangle_{10|F} + \langle ab\rangle_{11|F}\,,
\end{aligned}$$

where the conditional expectations are defined as $\langle ab\rangle_{xy|F} = \sum_{a,b} ab\, P(ab\,|\,xyF)$ with $P(ab\,|\,xyF)$ denoting the conditional probability distribution of ab given x, y and the values $F_1, ..., F_m$ of all factors.

If the random variables e^A, e^B are stochastically independent, each conditional expectation can be factorized given F, because

$$\begin{aligned}
\langle ab\rangle_{xy|F} &= \sum_{a,b} ab\, P(ab\,|\,xyF)\\
&= \sum_{a} aP(a\,|\,xF) \sum_{b} bP(b\,|\,yF)\\
&= \langle a\rangle_{x|F}\langle b\rangle_{y|F}\,.
\end{aligned} \tag{14}$$

Repeating the argument used at the end of the Proof of Proposition 1 we define four real numbers $a_0 := \langle a\rangle_{0|F}$, $a_1 := \langle a\rangle_{1|F}$, $b_0 := \langle b\rangle_{0|F}$ and $b_1 := \langle b\rangle_{1|F}$ which all lie in the interval $[-1, 1]$. This gives $S_{1|F} = a_0b_0+a_0b_1+a_1b_0-a_1b_1 = a_0(b_0 + b_1) + a_1(b_0 - b_1)$ by Equation (14) and hence $|S_{1|F}| \leq 2$. The proof of $|S_{i|F}| \leq 2$ for $i = 2, 3, 4$ is similar.

In the main part of the paper, we consider discrete distributions, which are appropriate from a practical standpoint as financial data is truncated to a certain number of digits after the decimal point. Otherwise, from a standpoint of mathematical finance theory, continuous distributions are assumed for random variables and factors F, so proper care is needed with Equation (14). Technically, Equation (14) should be understood as holding true on $\{F \in J\}$ for a small Borel subset J of $\mathbb{R}^m$. If J contains just one point and the probability distribution of the factors admit a Lebesgue density, then $P\{F \in J\} = 0$, so conditional probabilities cannot be defined by simply dividing by the probability of the event. They can, however, be defined almost surely with respect to P. If the probability density function is continuous, a small enough set J can be chosen such that the density is almost constant on J, allowing the probability in Equation (14) to be factorized as shown. □

Appendix A: Further proofs

Here we prove Propositions 2, 4 and 6.

We also give, in *Appendix B*, an explicit example of a Factor Model, such that Equation (10) is true, and explain the connection to Simpson's paradox. Finally, we show in *Appendix C* how Proposition 5 can be applied to larger ranges of factor values, provided that the probabilities for the measurement settings are stable within those ranges.

Proof of Proposition 2. By definition of the S-value in Equations (1)-(4) we have

$$\begin{aligned} S_1 &= \quad c + d + e - f\,, \\ S_2 &= \quad c + d - e + f\,, \\ S_3 &= \quad c - d + e + f\,, \\ S_4 &= -c + d + e + f\,, \end{aligned}$$

where the four real numbers c, d, e, f stand for the four expectation values and thus lie each in the interval $[-1; +1]$. Assuming without loss of generality that $|S_1| > 2$ as well as $|S_2| > 2$ we have that one of the following four cases has to hold:

- $S_1 > 2$ and $S_2 > 2$ and therefore $2c + 2d = S_1 + S_2 > 4$ contradicting $\{c, d\} \in [-1; +1]$.
- $S_1 > 2$ and $S_2 < -2$ and therefore $2e - 2f = S_1 - S_2 > 4$ contradicting $\{e, f\} \in [-1; +1]$.
- $S_1 < -2$ and $S_2 > +2$ and therefore $-2e + 2f = -S_1 + S_2 > 4$ contradicting $\{e, f\} \in [-1; +1]$.
- $S_1 < -2$ and $S_2 < -2$ and therefore $-2c - 2d = -S_1 - S_2 > 4$ contradicting $\{c, d\} \in [-1; +1]$.

Thus a contradiction ensues for all four cases, showing that the assumption cannot be true. This proves the proposition.□

Proof of Proposition 4, Part (i). To prove the first assertion (i), consider some arbitrary statistics $\tilde{P}(a, b, x, y)$. We will need to specify an explicit structural causal model [13] compatible with the Model (b) in Fig. 1. It is supposed to reproduce the statistic $\tilde{P}(a, b, x, y)$ with the dichotomous variables U_1 and U_2. In addition, we shall avail ourselves of an extra error term θ affecting only the variables a and b. See Fig. 4 for an illustration.

We observe that by Bayes' rule we can decompose the distribution $\tilde{P}(a, b, x, y)$ in the following way

$$\tilde{P}(a, b, x, y) = \tilde{P}(a, b|x, y)\,\tilde{P}(x, y)\,, \tag{A1}$$

where $\tilde{P}(a, b|x, y)$ is the ususal conditional probability and $\tilde{P}(x, y) = \sum_{a,b} \tilde{P}(a, b, x, y)$.

Let us denote components of the variables U_1 and U_2 as follows

$$U_1 \equiv (x_1, y_1)\,, \tag{A2}$$

$$U_2 \equiv (x_2, y_2)\,, \tag{A3}$$

with dichotomous variables $x_1, x_2, y_1, y_2 = 0, 1$. We postulate a trivial relation between those variables

$$U_2 := U_1 \qquad \text{(i.e., } x_2 := x_1 \text{ and } y_2 := y_1)\,, \tag{A4}$$

specifying the arrow $U_1 \to U_2$. Then, we define the first part of the structural causal model in the following way

$$x := x_1\,, \tag{A5}$$

$$y := y_1\,, \tag{A6}$$

which describes the respective arrows $U_1 \to x$ and $U_1 \to y$. Furthermore, we set the distribution of U_1 in the form

$$P(U_1) \equiv P(x_1, y_1) \;:=\; \tilde{P}(x_1, y_1)\,, \tag{A7}$$

where, as above, we use the definition $\tilde{P}(x_1, y_1) = \sum_{a,b} \tilde{P}(a, b, x_1, y_1)$.

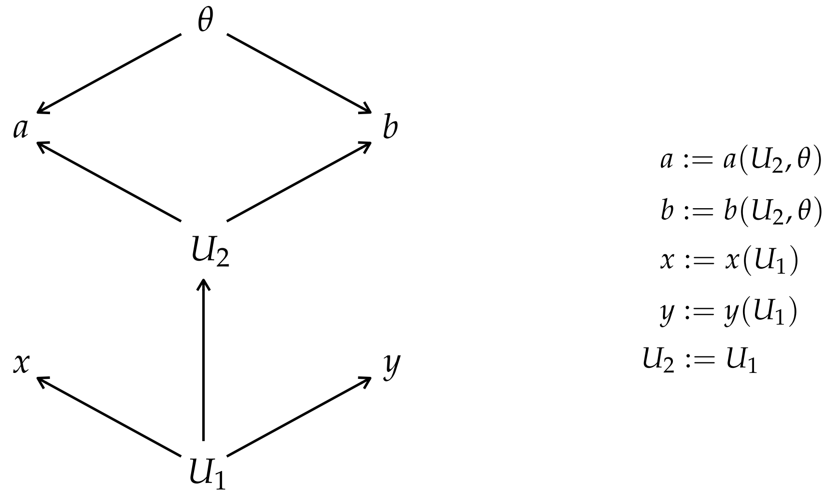

FIG. 4. A graphical representation of the structural causal model used in the proof of part (i) of Proposition 4.

Now, consider an error term θ, common to a, b, which is uniformly distributed over the unit interval $[0, 1]$. Furthermore, we define a set of characteristic functions $\chi_{ab}^{(x,y)}$ for $a, b = \pm 1$ as follows

$$\chi_{--}^{(x,y)}(\theta) := \begin{cases} 1\,, & \text{if} \quad 0 \leqslant \theta \leqslant \tilde{P}(-,-|x,y)\,, \\ 0\,, & \text{otherwise,} \end{cases} \tag{A8}$$

$$\chi_{-+}^{(x,y)}(\theta) := \begin{cases} 1\,, & \text{if} \quad \tilde{P}(-,-|x,y) < \theta \leqslant \tilde{P}(-,-|x,y) + \tilde{P}(-,+|x,y)\,, \\ 0\,, & \text{otherwise,} \end{cases} \tag{A9}$$

$$\chi_{+-}^{(x,y)}(\theta) := \begin{cases} 1\,, & \text{if} \quad \tilde{P}(-,-|x,y) + \tilde{P}(-,+|x,y) < \theta \leqslant \tilde{P}(-,-|x,y) + \tilde{P}(-,+|x,y) + \tilde{P}(+,-|x,y)\,, \\ 0\,, & \text{otherwise,} \end{cases} \tag{A10}$$

$$\chi_{++}^{(x,y)}(\theta) := \begin{cases} 1\,, & \text{if} \quad \tilde{P}(-,-|x,y) + \tilde{P}(-,+|x,y) + \tilde{P}(+,-|x,y) < \theta \leqslant 1\,, \\ 0\,, & \text{otherwise,} \end{cases} \tag{A11}$$

where the conditional distribution $\tilde{P}(a, b|x, y)$ is used as defined in Equation (A1). Support of those functions is graphically depicted here:

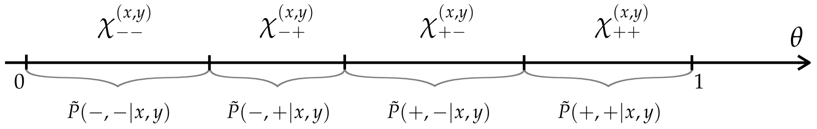

Note that all $\chi_{ab}^{(x,y)}$ are non-overlapping for different $a, b = \pm 1$ (when $x, y = 0, 1$ are fixed). Since θ is uniformly distributed, it is straightforward to observe that

$$\int P\big[\,\chi_{ab}^{(x,y)}(\theta) = 1\,\big]\, P(\theta)\, d\theta = \tilde{P}(a, b|x, y) \qquad \text{for each} \;\; a, b, x, y. \tag{A12}$$

This allows to define the remaining part of the structural causal model as follows

$$a := 2\left[\,\chi_{+-}^{(x_2,y_2)}(\theta) + \chi_{++}^{(x_2,y_2)}(\theta)\right] - 1, \tag{A13}$$

$$b := 2\left[\,\chi_{-+}^{(x_2,y_2)}(\theta) + \chi_{++}^{(x_2,y_2)}(\theta)\right] - 1, \tag{A14}$$

which specify respectively the arrows $U_2 \to a \leftarrow \theta$ and $U_2 \to b \leftarrow \theta$. After closer inspection, such defined variables a and b when combined together lend themselves to a simple characterisation:

$$(a, b) = (\alpha, \beta) \Leftrightarrow \chi_{\alpha\beta}^{(x_2,y_2)}(\theta) = 1, \tag{A15}$$

where $\alpha, \beta = \pm 1$ are possible values taken by the respective variables a, b.

Compared to Model (b) in Fig. 1 the structural causal model, as defined in Equations (A2)-(A6) and Equations (A13)-(A14), and shown graphically in Fig. 4, is more restrictive in the sense that it does not need arrows $x \to a$ and $y \to b$. Note that the causal model shown in Fig. 4 shows an independent error term θ which affects variables a and b, so that the variables a, b, describing (market) direction, depend on both θ and U_2. It is interesting to observe that the lower and the upper part of the diagram in Fig. 4, i.e. variables x, y, U_1 and variables a, b and U_2 respectively, are mediated with a single arrow $U_1 \to U_2$. In other words the variables x, y and a, b are partly synchronized through the process described by the link between the dichotomous variables U_1, U_2.

It remains to show that the joint probability distribution $P(a,b,x,y)$ generated by the above model reproduces the desired statistic $\tilde{P}(a,b,x,y)$. From the graph structure shown in Fig. 4, we can write the joint distribution in a product form and then use the specifics of the structural equations defining the model

$$\begin{aligned}
P(a,b,x,y) &= \sum_{U_1,U_2} \int P(a|U_2,\theta)\, P(b|U_2,\theta)\, P(U_2|U_1)\, P(x|U_1)\, P(y|U_1)\, P(U_1)\, P(\theta)\, d\theta && \text{(A16)}\\
&\overset{\text{(A2)(A5)(A7)}}{=} \sum_{U_2} \int P(a|U_2,\theta)\, P(b|U_2,\theta)\, P(U_2|U_1=(x,y))\, \tilde{P}(x,y)\, P(\theta)\, d\theta && \text{(A17)}\\
&\overset{\text{(A4)}}{=} \int P(a|U_2=(x,y),\theta)\, P(b|U_2=(x,y),\theta)\, \tilde{P}(x,y)\, P(\theta)\, d\theta && \text{(A18)}\\
&\overset{\text{(A15)}}{=} \int P\big[\, \chi_{ab}^{(x,y)}(\theta)=1\,\big]\, \tilde{P}(x,y)\, P(\theta)\, d\theta && \text{(A19)}\\
&\overset{\text{(A12)}}{=} \tilde{P}(a,b|x,y)\, \tilde{P}(x,y) && \text{(A20)}\\
&\overset{\text{(A1)}}{=} \tilde{P}(a,b,x,y)\,. && \text{(A21)}
\end{aligned}$$

We note that the reduction from Equation (A17) to Equation (A19) relies on the definitions in Equations (A13) and (A14), but also on the fact that $\chi_{ab}^{(x,y)}$ are non-overlapping for different a,b. Hence, we have proved that the model constructed above does indeed reproduce the desired statistic $\tilde{P}(a,b,x,y)$, which shows Assertion (i) of Proposition 4.

Proof of Proposition 4, Part (ii). To prove the second assertion (*ii*) we will provide a full parametric model. For illustrative purposes, the parametrization is more general than what would be needed just for a proof of assertion (*ii*). We assume two free parameters γ and ϵ, which may take any value in the interval $[0,1]$. Parameter γ is used for the conditional probability of U_2 given cause U_1, whereas parameter ϵ is used to describe the probability for a,b given a value for cause U_2. The probability for x,y is solely modelled via cause U_1 while the probability for a,b is solely modelled by cause U_2.

Based on these two parameters we will give a specific form for the probability distribution of the bivalued unknown causes in Model (b), which allows us to compute the expectation values $\langle ab\rangle_{xy}$ for the four cases $xy = 11,\, 10,\, 01,\, 00$ and show

$$S_1 = 4(1-2\gamma)(1-2\epsilon) \text{ and } S_2 = S_3 = S_4 = 0. \tag{A22}$$

Equation (A22) is illustrated in Fig. 5. The parameter γ may be interpreted as the connection strength between the common causes U_1 and U_2, while the parameter ϵ describes the distribution of outcomes a,b under different regimes.

Specifically, we may choose $\epsilon = 0$, so Equation (A22) reduces to $S_1 = 4(1-2\gamma)$, which implies that S_1 can take any value in the interval $[-4,+4]$. So, one parameter $\gamma \in [0,1]$ is sufficient to generate every possible S_1-value.

Specification of the model: We define the probability distribution of the bivalued unknown causes U_1 and U_2 by

$$P(U_2\,|U_1) := \begin{pmatrix} \gamma & 1-\gamma \\ 1-\gamma & \gamma \end{pmatrix} \tag{A23}$$

and

$$P(a,b\,|U_2=-1) := \frac{1}{2}\begin{pmatrix} 1-\epsilon & \epsilon \\ \epsilon & 1-\epsilon \end{pmatrix}, \tag{A24}$$

$$P(a,b\,|U_2=+1) := \frac{1}{2}\begin{pmatrix} \epsilon & 1-\epsilon \\ 1-\epsilon & \epsilon \end{pmatrix}. \tag{A25}$$

Furthermore, we stipulate

$$P(U_1=-1) = \frac{1}{4},\; P(U_1=+1) = \frac{3}{4} \tag{A26}$$

and

$$P(xy=00\,|\,U_1=-1) = P(xy=01\,|\,U_1=-1) = P(xy=10\,|\,U_1=-1) = 0,\; P(xy=11\,|\,U_1=-1) = 1, \tag{A27}$$

$$P(xy=00\,|\,U_1=+1) = P(xy=01\,|\,U_1=+1) = P(xy=10\,|\,U_1=+1) = \frac{1}{3},\; P(xy=11\,|\,U_1=+1) = 0, \tag{A28}$$

which means that the setting $xy = 11$ occurs if and only if $U_1 = -1$, whereas in the case of $U_1 = 1$ the three cases $xy = 00, 10, 01$ are all equally likely, while $xy = 11$ is impossible.

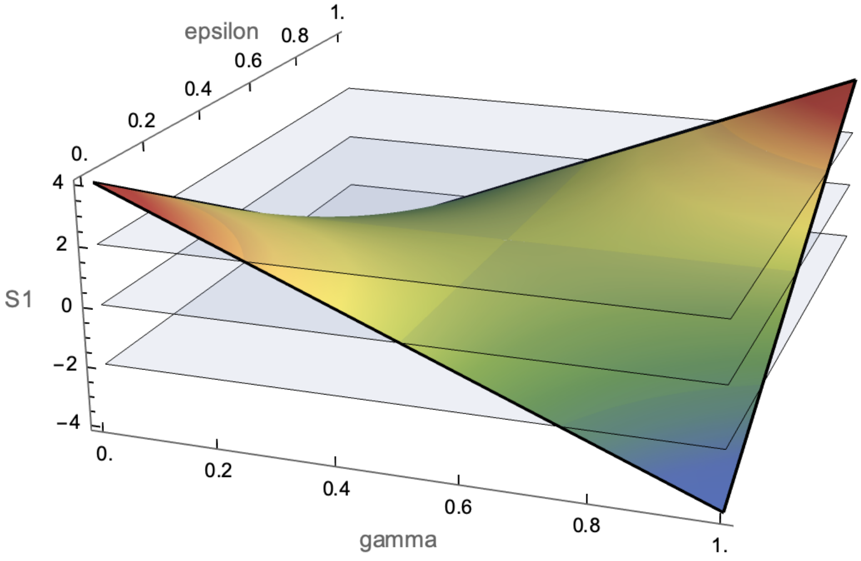

FIG. 5. S_1-values shown as a function of γ and ϵ for the parametric model specified in the proof of Proposition 4. It can be clearly seen that there are many combinations of the parameters γ, ϵ, for which S_1 is outside the range of $[-2, 2]$.

Finally, we assume for simplicity that there are no direct links links from x to a and from y to b in the causal diagram for Model (b).

Computation of the S-values: Equation (A24) implies

$$\sum_{a,b} ab\, P(ab \,|\, U_2 = -1) = (-1)^2(1-\epsilon)/2 + (-1)(+1)\epsilon/2 + (+1)(-1)\epsilon/2 + (1-\epsilon)/2 = 1 - 2\epsilon,$$

and similarly Equation (A25) implies

$$\sum_{a,b} ab\, P(ab \,|\, U_2 = +1) = (-1)^2\epsilon/2 + (-1)(+1)(1-\epsilon)/2 + (+1)(-1)(1-\epsilon)/2 + \epsilon/2 = 2\epsilon - 1.$$

The S-values are derived from the expectation values $\langle ab \rangle_{xy} = \sum_{a,b} ab\, P(ab \,|\, xy)$ using Equations (1)-(4). The Bayesian network shown in Model (b) of Fig. 1 gives the following factorization of the joint probability distribution

$$P(a, b, x, y, U_2, U_1) = P(a, b \,|\, x, y, U_2) P(U_2 \,|\, U_1) P(x, y \,|\, U_1) P(U_1).$$

With the assumption that there are no direct links from x, y to a, b, this joint probability distribution simplifies to

$$P(a, b, x, y, U_2, U_1) = P(a, b \,|\, U_2) P(U_2 \,|\, U_1) P(x, y \,|\, U_1) P(U_1).$$

With this we can now simplify the generic expression for the expectation values

$$\langle ab \rangle_{xy} = \sum_{a,b} ab\, P(ab \,|\, xy) = \sum_{a,b} ab\, \frac{P(a, b, x, y)}{P(x, y)} = \sum_{a,b} ab \frac{\sum_{U_2, U_1} P(a, b, x, y, U_2, U_1)}{\sum_{U_1} P(x, y, U_1)}$$
$$= \sum_{a,b} ab \frac{\sum_{U_2, U_1} P(a, b \,|\, U_2) P(U_2 \,|\, U_1) P(x, y \,|\, U_1) P(U_1)}{\sum_{U_1} P(x, y \,|\, U_1) P(U_1)}$$

for the four cases $xy = 00$, $xy = 01$, $xy = 10$, $xy = 11$.

The case $xy = 11$: This case corresponds to $U_1 = -1$. We now use the fact that Equation (A23) implies the following conditional probabilities

$$P(U_2 = -1 \,|\, U_1 = -1) = \gamma,$$
$$P(U_2 = +1 \,|\, U_1 = -1) = 1 - \gamma.$$

With this we compute

$$
\begin{aligned}
\langle ab\rangle_{11} &= \sum_{a,b} ab\, P(ab\,|\,11) = \sum_{a,b} ab\, \frac{P(a,b,x=1,y=1)}{P(xy=11)} = \sum_{a,b} ab \frac{\sum_{U_2,U_1} P(a,b\,|\,U_2)P(U_2\,|\,U_1)P(xy=11\,|\,U_1)P(U_1)}{\sum_{U_1} P(xy=11\,|\,U_1)P(U_1)} \\
&= \sum_{a,b} ab \frac{\sum_{U_2} P(a,b\,|\,U_2)P(U_2\,|\,U_1=-1)P(U_1=-1)}{1\cdot 1/4} \\
&= 4\sum_{a,b} ab\,(P(a,b\,|\,U_2=-1)P(U_2=-1\,|\,U_1=-1)P(U_1=-1) + P(a,b\,|\,U_2=1)P(U_2=1\,|\,U_1=-1)P(U_1=-1)) \\
&= 4\sum_{a,b} ab\left(P(a,b\,|\,U_2=-1)\gamma\frac{1}{4} + P(a,b\,|\,U_2=1)(1-\gamma)\frac{1}{4}\right) \\
&= \gamma\sum_{a,b} abP(a,b\,|\,U_2=-1) + (1-\gamma)\sum_{a,b} abP(a,b\,|\,U_2=1) \\
&= \gamma(1-2\epsilon) + (1-\gamma)(2\epsilon-1) \\
&= -(1-2\gamma)(1-2\epsilon).
\end{aligned}
$$

The case $xy = 10$: This case corresponds to $U_1 = 1$. We now use the fact that Equation (A23) implies the following conditional probabilities

$$
\begin{aligned}
P(U_2 = -1\,|\,U_1 = +1) &= 1-\gamma, \\
P(U_2 = +1\,|\,U_1 = +1) &= \gamma.
\end{aligned}
$$

With this we compute

$$
\begin{aligned}
\langle ab\rangle_{10} &= \sum_{a,b} ab\, P(ab\,|\,10) = \sum_{a,b} ab\, \frac{P(a,b,x=1,y=0)}{P(xy=10)} = \sum_{a,b} ab \frac{\sum_{U_2,U_1} P(a,b\,|\,U_2)P(U_2\,|\,U_1)P(xy=10\,|\,U_1)P(U_1)}{\sum_{U_1} P(xy=10\,|\,U_1)P(U_1)} \\
&= \sum_{a,b} ab \frac{\sum_{U_2} P(a,b\,|\,U_2)P(U_2\,|\,U_1=1)P(xy=10\,|\,U_1=1)P(U_1=1)}{1/3\cdot 3/4} \\
&= 4\sum_{a,b} ab \sum_{U_2} P(a,b\,|\,U_2)P(U_2\,|\,U_1=1)P(xy=10\,|\,U_1=1)P(U_1=1) \\
&= 4\sum_{a,b} ab\left(P(a,b\,|\,U_2=-1)P(U_2=-1\,|\,U_1=1)\cdot\frac{1}{3}\cdot\frac{3}{4} + P(a,b\,|\,U_2=1)P(U_2=1\,|\,U_1=1)\cdot\frac{1}{3}\cdot\frac{3}{4}\right) \\
&= \sum_{a,b} ab\,(P(a,b\,|\,U_2=-1)(1-\gamma) + P(a,b\,|\,U_2=1)\gamma) \\
&= (1-\gamma)\sum_{a,b} abP(a,b\,|\,U_2=-1) + \gamma\sum_{a,b} abP(a,b\,|\,U_2=1) \\
&= (1-\gamma)(1-2\epsilon) + \gamma(2\epsilon-1) \\
&= (1-2\gamma)(1-2\epsilon).
\end{aligned}
$$

The case $xy = 01$: This case corresponds to $U_1 = 1$ and can be treated similarly to the previous one. We again use the fact that Equation (A23) implies the following conditional probabilities

$$
\begin{aligned}
P(U_2 = -1\,|\,U_1 = +1) &= 1-\gamma, \\
P(U_2 = +1\,|\,U_1 = +1) &= \gamma.
\end{aligned}
$$

With this we compute

$$\begin{aligned}
\langle ab\rangle_{01} &= \sum_{a,b} ab\, P(ab\,|\,01) = \sum_{a,b} ab\,\frac{P(a,b,x=0,y=1)}{P(xy=01)} = \sum_{a,b} ab\frac{\sum_{U_2,U_1} P(a,b\,|\,U_2)P(U_2\,|\,U_1)P(xy=01\,|\,U_1)P(U_1)}{\sum_{U_1} P(xy=01\,|\,U_1)P(U_1)}\\
&= \sum_{a,b} ab\frac{\sum_{U_2} P(a,b\,|\,U_2)P(U_2\,|\,U_1=1)P(xy=01\,|\,U_1=1)P(U_1=1)}{1/3\cdot 3/4}\\
&= 4\sum_{a,b} ab\sum_{U_2} P(a,b\,|\,U_2)P(U_2\,|\,U_1=1)P(xy=01\,|\,U_1=1)P(U_1=1)\\
&= 4\sum_{a,b} ab\left(P(a,b\,|\,U_2=-1)P(U_2=-1\,|\,U_1=1)\cdot\frac{1}{3}\cdot\frac{3}{4} + P(a,b\,|\,U_2=1)P(U_2=1\,|\,U_1=1)\cdot\frac{1}{3}\cdot\frac{3}{4}\right)\\
&= \sum_{a,b} ab\,(P(a,b\,|\,U_2=-1)(1-\gamma) + P(a,b\,|\,U_2=1)\gamma)\\
&= (1-\gamma)\sum_{a,b} abP(a,b\,|\,U_2=-1) + \gamma\sum_{a,b} abP(a,b\,|\,U_2=1)\\
&= (1-\gamma)(1-2\epsilon) + \gamma(2\epsilon-1)\\
&= (1-2\gamma)(1-2\epsilon).
\end{aligned}$$

The case $xy = 00$: This case corresponds to $U_1 = 1$ and can be treated similarly to the two previous ones yielding again

$$\langle ab\rangle_{00} = (1-2\gamma)(1-2\epsilon).$$

With the abbreviation $\varphi := (1-2\gamma)(1-2\epsilon)$ the results of the four cases can be summarized as

$$\langle ab\rangle_{11} = -\varphi,\ \langle ab\rangle_{10} = \langle ab\rangle_{01} = \langle ab\rangle_{00} = \varphi.$$

Together with the definiton of S_1 in Equation (1) we obtain

$$S_1 = \langle ab\rangle_{00} + \langle ab\rangle_{01} + \langle ab\rangle_{10} - \langle ab\rangle_{11} = \varphi+\varphi+\varphi-(-\varphi) = 4\varphi.$$

From Equations (2) to (4) we get

$$\begin{aligned}
S_2 &= \langle ab\rangle_{00} + \langle ab\rangle_{01} - \langle ab\rangle_{10} + \langle ab\rangle_{11} = \varphi+\varphi-\varphi+(-\varphi) = 0,\\
S_3 &= \langle ab\rangle_{00} - \langle ab\rangle_{01} + \langle ab\rangle_{10} + \langle ab\rangle_{11} = \varphi-\varphi+\varphi+(-\varphi) = 0,\\
S_3 &= -\langle ab\rangle_{00} + \langle ab\rangle_{01} + \langle ab\rangle_{10} + \langle ab\rangle_{11} = -\varphi+\varphi+\varphi+(-\varphi) = 0.
\end{aligned}$$

This proves Equation (A22) and thus Proposition 4. □

Remark 1 *The explicit minimalistic model specified by Equations (A23) to (A28) was given as a point of principle to show that it is possible to generate the full range of values that are algebraically possible for S_1 in a simple way. This stands in contrast to the Tsirelson bound of $2\sqrt{2}$ for the S_1-value in quantum theory.*

As constructed above, the simple model leads to values of zero for S_2, S_3 and S_4, which is not realistic when working with real data. However, the model can be easily generalised to yield non-zero numbers for all four S_i-values. This can be achieved by introducing additional free parameters to break the symmetry in Equations (A23) to (A25) and for setting the values of the probabilities in Equations (A26) to (A28).

Proof of Proposition 6. If the joint price return (R^A, R^B) of two securities follows the bivariate Gaussian model in Equations (11) to (12) and if the thresholds that separate weak from strong days are given as $r_A = \alpha\sigma_A$ and $r_B = \beta\sigma_B$ with positive constants α, β, then all four expectation values that make up the definition of S_1 follow from the bivariate Gaussian density

$$p_\rho(v,w) = \frac{\exp\left(-\frac{1}{2(1-\rho^2)}\left(\frac{v^2}{\sigma_A^2}+\frac{w^2}{\sigma_B^2}-2\frac{\rho v w}{\sigma_A\sigma_B}\right)\right)}{2\pi\sigma_A\sigma_B\sqrt{1-\rho^2}}.$$

This density has the symmetry properties $p_\rho(v,w) = p_\rho(-v,-w) = p_{-\rho}(v,-w) = p_{-\rho}(-v,w)$ yielding

$$\begin{aligned}
\langle ab\rangle_{00} &= \mathbb{E}\left[\text{sign}(R^A)\text{sign}(R^B)1_{|R^A|>r_A}1_{|R^B|>r_B}\right] = \int_{-\infty}^{\infty}\int_{-\infty}^{\infty}\text{sign}(v)\text{sign}(w)1_{|v|>r_A}1_{|w|>r_B}p_\rho(v,w)dvdw\\
&= \frac{\int_{r_A}^{\infty}\int_{r_B}^{\infty}(p_\rho(v,w)-p_{-\rho}(v,w))\,dvdw}{\int_{r_A}^{\infty}\int_{r_B}^{\infty}(p_\rho(v,w)+p_{-\rho}(v,w))\,dvdw} = \frac{\int_{\gamma_x}^{\infty}\int_{\gamma_y}^{\infty}e^{-\tilde{v}^2-\tilde{w}^2}\sinh(2\rho\tilde{v}\tilde{w})d\tilde{v}d\tilde{w}}{\int_{\gamma_x}^{\infty}\int_{\gamma_y}^{\infty}e^{-\tilde{v}^2-\tilde{w}^2}\cosh(2\rho\tilde{v}\tilde{w})d\tilde{v}d\tilde{w}}
\end{aligned}$$

with the substitution $\tilde{v} = \frac{\gamma_x}{r_A} v, \tilde{w} = \frac{\gamma_x}{r_B} w$ where

$$\gamma_x = \frac{\alpha}{\sqrt{2(1-\rho^2)}},\ \gamma_y = \frac{\beta}{\sqrt{2(1-\rho^2)}}.$$

Fortunately, both sinh and cosh have a series expansion that is convergent for every argument z

$$\sinh(z) = \sum_{n=0}^{\infty} \frac{z^{2n+1}}{(2n+1)!},\ \cosh(z) = \sum_{n=0}^{\infty} \frac{z^{2n}}{(2n)!},$$

which allows writing the above integrals as an infinite series, reducing them to the incomplete gamma functions

$$\langle ab\rangle_{00} = \frac{\sum_{n=0}^{\infty} c_{2n+1}\Gamma(n+1,\gamma_x^2)\Gamma(n+1,\gamma_y^2)}{\sum_{n=0}^{\infty} c_{2n}\Gamma(n+1/2,\gamma_x^2)\Gamma(n+1/2,\gamma_y^2)}$$

with the definition $c_k = 2^k\rho^k/k!$. The other expectation values $\langle ab\rangle_{01}, \langle ab\rangle_{10}, \langle ab\rangle_{11}$, have analytic expressions that can be computed analogously

$$\langle ab\rangle_{10} = \frac{\int_0^{\gamma_x}\int_{\gamma_y}^{\infty} e^{-\tilde{v}^2-\tilde{w}^2}\sinh(2\rho\tilde{v}\tilde{w})d\tilde{v}d\tilde{w}}{\int_0^{\gamma_x}\int_{\gamma_y}^{\infty} e^{-\tilde{v}^2-\tilde{w}^2}\cosh(2\rho\tilde{v}\tilde{w})d\tilde{v}d\tilde{w}} = \frac{\sum_{n=0}^{\infty} c_{2n+1}(\Gamma(n+1,0)-\Gamma(n+1,\gamma_x^2))\Gamma(n+1,\gamma_y^2)}{\sum_{n=0}^{\infty} c_{2n}(\Gamma(n+1/2,0)-\Gamma(n+1/2,\gamma_x^2))\Gamma(n+1/2,\gamma_y^2)},$$

$$\langle ab\rangle_{01} = \frac{\int_{\gamma_x}^{\infty}\int_0^{\gamma_y} e^{-\tilde{v}^2-\tilde{w}^2}\sinh(2\rho\tilde{v}\tilde{w})d\tilde{v}d\tilde{w}}{\int_{\gamma_x}^{\infty}\int_0^{\gamma_y} e^{-\tilde{v}^2-\tilde{w}^2}\cosh(2\rho\tilde{v}\tilde{w})d\tilde{v}d\tilde{w}} = \frac{\sum_{n=0}^{\infty} c_{2n+1}(\Gamma(n+1,0)-\Gamma(n+1,\gamma_y^2))\Gamma(n+1,\gamma_x^2)}{\sum_{n=0}^{\infty} c_{2n}(\Gamma(n+1/2,0)-\Gamma(n+1/2,\gamma_y^2))\Gamma(n+1/2,\gamma_x^2)},$$

$$\langle ab\rangle_{11} = \frac{\int_0^{\gamma_x}\int_0^{\gamma_y} e^{-\tilde{v}^2-\tilde{w}^2}\sinh(2\rho\tilde{v}\tilde{w})d\tilde{v}d\tilde{w}}{\int_0^{\gamma_x}\int_0^{\gamma_y} e^{-\tilde{v}^2-\tilde{w}^2}\cosh(2\rho\tilde{v}\tilde{w})d\tilde{v}d\tilde{w}} = \frac{\sum_{n=0}^{\infty} c_{2n+1}(\Gamma(n+1,0)-\Gamma(n+1,\gamma_x^2))(\Gamma(n+1,0)-\Gamma(n+1,\gamma_y^2))}{\sum_{n=0}^{\infty} c_{2n}(\Gamma(n+1/2,0)-\Gamma(n+1/2,\gamma_x^2))(\Gamma(n+1/2,0)-\Gamma(n+1/2,\gamma_y^2))}.$$

The combination of the four expected value terms above lead to an analytic formula for S_1 in the Gaussian model

$$S_1^{\text{Gauss}} = \langle ab\rangle_{00} + \langle ab\rangle_{01} + \langle ab\rangle_{10} - \langle ab\rangle_{11}.$$

The other quantities $S_2^{\text{Gauss}}, S_3^{\text{Gauss}}, S_4^{\text{Gauss}}$ can be easily obtained from the same four expected value terms. □

Appendix B: Simpson's paradox - a cautionary example

We will give an explicit example to show that it is possible to have Equation (10), i.e.

$$S_1 \neq \sum_F S_{1|F}\, P(F), \tag{B1}$$

in the situation of a Factor Model as defined by Equations (8) and (9). Details will be given in the subsequent proof. One purpose of this example is to warn against taking a shortcut to calculate S-values using its conditional version, in analogy with the law of total probability.

It is important to keep Equation (B1) in mind when dealing with conditional S-values, because if it were possible to proceed with the computation of S_1 by analogy with the law of total probability, then $S_{1|F} \leqslant 2$ for all factor values would imply $S_1 \leqslant 2$. So from Proposition 5 it would follow that Factor Models generally have the property $S_1 \leqslant 2$, which is not the case.

This surprising phenomenon is evocative of a case of Simpson's paradox [62, 63] where some characteristic of interest radically changes behaviour, when considering some conditional distribution of the characteristic vs. its unconditional distribution.

Proof. We want to construct an explicit Factor Model satisfying (B1). First, let us recall the definition of the quantity of interest:

$$S_1 = \langle ab\rangle_{00} + \langle ab\rangle_{01} + \langle ab\rangle_{10} - \langle ab\rangle_{11}, \tag{B2}$$

where $\langle ab\rangle_{xy} := \sum_{a,b} ab\, P(ab|xy)$ is the correlation function. By conditioning on F, we can write the latter quantity as follows

$$\langle ab\rangle_{xy} = \sum_F \sum_{a,b} ab\, P(ab|xy)\, P(F|xy) = \sum_F \langle ab\rangle_{xyF}\, P(F|xy), \tag{B3}$$

where $\langle ab\rangle_{xyF} := \sum_{a,b} ab\, P(ab|xyF)$ denotes the correlation function conditioned on F. Please note that here, as in the main part, we have used the compact notation $\sum_{a,b} ab\, P(ab|xyF)$ where we mean $\sum_{a,b} ab\, P(a,b|x,y,F)$ to avoid a heavy use of commas. Clearly, we can also define the S-value conditioned on F (calculated for data restricted to a specific value of F).

$$S_{1|F} = \langle ab\rangle_{00F} + \langle ab\rangle_{01F} + \langle ab\rangle_{10F} - \langle ab\rangle_{11F}, \tag{B4}$$

We now offer a counterintuitive example to illustrate the surprising phenomenon that in the factor model defined in Equations (8) and (9) and shown in Figure 2, the S-value can exceed 2 while conditionalising on F it is always less then 2 (as shown in Proposition 5). To see this we will construct an example in which

$$S_1 > 2 \quad \text{and} \quad S_{1|F} \leqslant 2 \;\; \text{for all } F. \tag{B5}$$

Consider a random variable F taking two possible values $F = 1, 2$ with equal probability

$$P(F = 1) = P(F = 2) = {}^{1}\!/_{2}\,. \tag{B6}$$

Now, let R_A and R_B be random variables with four possible values $R_A, R_B = \pm 1, \pm 2$, defined in accordance with Equations (8) and (9) in the following way

$$P(R_A = +1|F = 1) = {}^{1}\!/_{2}, \qquad P(R_A = +2|F = 1) = {}^{1}\!/_{2}, \tag{B7}$$
$$P(R_A = -1|F = 2) = {}^{2}\!/_{3}, \qquad P(R_A = +2|F = 2) = {}^{1}\!/_{3}, \tag{B8}$$

and

$$P(R_B = +1|F = 1) = {}^{1}\!/_{2}, \qquad P(R_B = +2|F = 1) = {}^{1}\!/_{2}, \tag{B9}$$
$$P(R_B = +1|F = 2) = {}^{1}\!/_{3}, \qquad P(R_B = +2|F = 2) = {}^{2}\!/_{3}. \tag{B10}$$

The variables x, y, and a, b be defined as we always do it in this paper

$$x := 1_{|R_A| \leqslant r_A},\; y := 1_{|R_B| \leqslant r_B},\; a := \text{sign}(R_A),\; b := \text{sign}(R_B). \tag{B11}$$

Then, from Equations (B6) and (B7)-(B10), for the threshold $r_A = r_B = {}^{3}\!/_{2}$ we get the conditional distribution of x, y as follows

$$P(xy|F = 1) = {}^{1}\!/_{4} \text{ for all } xy = 00, 01, 10, 11 \tag{B12}$$

and

$$P(xy|F = 2) = {}^{1}\!/_{9} \text{ for } xy = 00, \tag{B13}$$
$$P(xy|F = 2) = {}^{2}\!/_{9} \text{ for } xy = 01 \text{ and } 10, \tag{B14}$$
$$P(xy|F = 2) = {}^{4}\!/_{9} \text{ for } xy = 11, \tag{B15}$$

because R_A, R_B are independent conditional on F. Observe that these distributions differ for different values of $F = 1, 2$. Furthermore, we can calculate the conditional distribution of a, b which takes the form

$$P(a = +1, b = +1|xy, F = 1) = 1 \text{ for all } xy = 00, 01, 10, 11,$$

and

$$P(a = +1, b = +1|xy, F = 2) = 1 \text{ for } xy = 00 \text{ and } 01,$$
$$P(a = -1, b = +1|xy, F = 2) = 1 \text{ for } xy = 10 \text{ and } 11.$$

This immediately gives

$$\langle ab \rangle_{xy,F=1} = +1 \text{ for all } xy = 00, 01, 10, 11, \tag{B16}$$

and

$$\langle ab \rangle_{xy,F=2} = +1 \text{ for } xy = 00 \text{ and } 01, \tag{B17}$$
$$\langle ab \rangle_{xy,F=2} = -1 \text{ for } xy = 10 \text{ and } 11, \tag{B18}$$

Thus, we can calculate the conditional S_1-value in Equation (B4)

$$S_{1|F=1} = 1 + 1 + 1 - 1 = 2 \text{ and } S_{1|F=2} = 1 + 1 + (-1) - (-1) = 2. \tag{B19}$$

Note that both conditional S-values are $S_{1|F} \leqslant 2$ in compliance with Proposition (5). Now we proceed to calculate the unconditional S-value in Equation (B2). To use the defining Equation (B3), we need to know the conditional probabilities $P(F|x, y)$. This can be obtained from Bayes' rule

$$P(F|x, y) = \frac{P(xy|F)\, P(F)}{\sum_F P(xy|F)\, P(F)},$$

noting that all the terms in this equation are already given in Equations (B6) and (B12)-(B15). The calculation reads as follows

$$\begin{aligned}
P(F = 1|xy = 00) &= \frac{{}^{1}\!/_{4} \cdot {}^{1}\!/_{2}}{{}^{1}\!/_{4} \cdot {}^{1}\!/_{2} + {}^{1}\!/_{9} \cdot {}^{1}\!/_{2}} = {}^{9}\!/_{13} \quad &\Rightarrow \quad P(F = 2|xy = 00) &= {}^{4}\!/_{13}, \\
P(F = 1|xy = 01) &= \frac{{}^{1}\!/_{4} \cdot {}^{1}\!/_{2}}{{}^{1}\!/_{4} \cdot {}^{1}\!/_{2} + {}^{2}\!/_{9} \cdot {}^{1}\!/_{2}} = {}^{9}\!/_{17} \quad &\Rightarrow \quad P(F = 2|xy = 01) &= {}^{8}\!/_{17}, \\
P(F = 1|xy = 10) &= \frac{{}^{1}\!/_{4} \cdot {}^{1}\!/_{2}}{{}^{1}\!/_{4} \cdot {}^{1}\!/_{2} + {}^{2}\!/_{9} \cdot {}^{1}\!/_{2}} = {}^{9}\!/_{17} \quad &\Rightarrow \quad P(F = 2|xy = 10) &= {}^{8}\!/_{17}, \\
P(F = 1|xy = 11) &= \frac{{}^{1}\!/_{4} \cdot {}^{1}\!/_{2}}{{}^{1}\!/_{4} \cdot {}^{1}\!/_{2} + {}^{4}\!/_{9} \cdot {}^{1}\!/_{2}} = {}^{9}\!/_{25} \quad &\Rightarrow \quad P(F = 2|xy = 11) &= {}^{16}\!/_{25}.
\end{aligned}$$

Those values, together with Equations (B16)-(B18), can be used in Equation (B3), which yields

$$\langle ab\rangle_{00} = 1\cdot\tfrac{9}{13} + 1\cdot\tfrac{4}{13} \;=\; 1,$$
$$\langle ab\rangle_{01} = 1\cdot\tfrac{9}{17} + 1\cdot\tfrac{8}{17} \;=\; 1,$$
$$\langle ab\rangle_{10} = 1\cdot\tfrac{9}{17} + (-1)\cdot\tfrac{8}{17} \;=\; 1/17,$$
$$\langle ab\rangle_{11} = 1\cdot\tfrac{9}{25} + (-1)\cdot\tfrac{16}{25} \;=\; -7/25,$$

Finally, we can calculate the unconditional S-value with Equation (B2)

$$S_1 = 1 + 1 + 1/17 - (-7/25) \;=\; 2\tfrac{144}{425} \;\approx 2.34 \;>\; 2.$$

The last equation together with Equation (B19) shows that the example has the desired property (B1). □

Remark 2 *The counterexample given in the above proof shows that we can have conditional S-values not exceeding 2 and still an unconditional S-value above 2, i.e.*

$$S_{1|F} \leqslant 2 \quad \text{for all } F \text{ (as in Proposition 5)} \;\not\Longrightarrow\; S_1 \leqslant 2. \tag{B20}$$

So, how is that possible? The intuition as to the validity of the implication in Equation (B20) comes from mistakenly thinking of (B1) as an equality. However, this is not possible, that is, it is not possible to have (B1) as an equality, because the S-value is not a probability – and so there is no analogy with the law of total probability.

It is interesting to observe that the construction of the above counterexample was possible due to differences in the conditional distribution $P(xy|F)$. Specifically, $P(xy|F=1) \neq P(xy|F=2)$, cf. Equation (B12) and Equations (B13)-(B15). Note, it appears that under the assumption of the same distribution $P(xy|F)$, for all F, the construction of an example of (B1) is not possible, since in this case we have

$$P(xy|F) = P(xy|F') \text{ for all } F, F' \quad\Longrightarrow\quad S_1 \;=\; \textstyle\sum_F S_{1|F}\, P(F). \tag{B21}$$

Under this assumption, we also have that: $S_{1|F} \leqslant 2$ for all F implies $S_1 \leqslant 2$. See Appendix C for the proof.

Remark 3 *As noted earlier, we observe that a situation where (B1) holds true is evocative of Simpson's paradox [62, 63]. The characteristics of the S-values seem to radically change their behaviour when considering a conditional distribution versus the full data. In our case the S-value is a figure of merit which decides the plausibility of the factor model assumption with the threshold $S = 2$. In the above example, we have conditional $S_{1|F} \leqslant 2$ while for the full data $S_1 > 2$. As it was noted, the reversal in the behaviour, $S_{1|F}$ versus S_1, stems from the difference in the distribution of the relevant variables x, y for different values of the conditioning factor variable F. Reversals in other examples of Simpson's paradox, come about in the same manner, i.e. due to the difference in the distribution of some relevant characteristics, for example regarding the severity of patients of a clinical condition across different treatments [13, 64] or regarding the returns achieved by active fund managers [65].*

Appendix C: Extending Proposition 5 to Intervals

Proposition 5 holds for $S_{1|F}$ computed from the data restricted to a particular value of F. This may be impractical if F takes a large set of values (possibly continuous), as the available statistics obtainable from given data sets may be insufficient if we restrict the sample to any single value of F. Here we show that Proposition 5 also holds for S-values computed over a larger range of F under the additional assumption of *stability* of the distribution $P(xy|F)$. Again, please note that $P(xy|F)$ is short notation for $P(x, y|F)$ to avoid the heavy use of commas.

Let $\mathcal{F}$ be any collection of disjoint sets of possible factor values. This may be a subset of the full range of values taken by the factor variable F. Then we define

$$S_{1|\mathcal{F}} := \langle ab\rangle_{00\mathcal{F}} + \langle ab\rangle_{01\mathcal{F}} + \langle ab\rangle_{10\mathcal{F}} - \langle ab\rangle_{11\mathcal{F}}, \tag{C1}$$

where $\langle ab\rangle_{xy\mathcal{F}} := \sum_{a,b} ab\, P_{\mathcal{F}}(ab|xy)$ is the correlation function computed with the restricted data in the subset $\mathcal{F}$. We consider a factor model as in Equations (8) and (9). Then, Proposition 5 extends to

Proposition 7 *If $P(xy|F) = P(xy|F')$ for all $F, F' \in \mathcal{F}$ and all $xy \in \{00, 01, 10, 11\}$, then $S_{1|\mathcal{F}} \leqslant 2$.*

Proof. Since $P_{\mathcal{F}}(ab|xy) = \sum_{F\in\mathcal{F}} P_{\mathcal{F}}(ab|xyF)\, P_{\mathcal{F}}(F|xy)$, we have

$$\langle ab\rangle_{xy\mathcal{F}} = \sum_{a,b} ab\, P_{\mathcal{F}}(ab|xy) \;=\; \sum_{a,b}\sum_{F\in\mathcal{F}} ab\, P_{\mathcal{F}}(ab|xyF)\, P_{\mathcal{F}}(F|xy).$$

By assumption x, y are independent of F for all $F \in \mathcal{F}$, that is $P_{\mathcal{F}}(xy|F) = P_{\mathcal{F}}(xy)$. Because the independence relation is symmetrical, then F is also independent from x, y for all $F \in \mathcal{F}$, which means that $P_{\mathcal{F}}(F|x, y) = P_{\mathcal{F}}(F)$. Therefore, we can write

$$\langle ab\rangle_{xy\mathcal{F}} = \sum_{F\in\mathcal{F}}\sum_{a,b} ab\, P_{\mathcal{F}}(ab|xyF)\, P_{\mathcal{F}}(F) \;=\; \sum_{F\in\mathcal{F}} \langle ab\rangle_{xyF}\, P_{\mathcal{F}}(F).$$

With this expression substituted into Equation (C1) we get

$$S_{1|\mathcal{F}} = \sum_{F\in\mathcal{F}} \Big[\langle ab\rangle_{00,F} + \langle ab\rangle_{01,F} + \langle ab\rangle_{10,F} - \langle ab\rangle_{11,F}\Big] P_{\mathcal{F}}(F) \;=\; \sum_{F\in\mathcal{F}} S_{1|F}\, P_{\mathcal{F}}(F) \;\leqslant\; \sum_{F\in\mathcal{F}} 2\, P_{\mathcal{F}}(F) \;\leqslant 2 \quad \text{(C2)}$$

where we have used $S_{1|F} \leqslant 2$ from Proposition 5. □

In conclusion, we note that in the last Equation (C2) we have also proved

Corollary 1 *If $P(xy|F) = P(xy|F')$ for all $F, F' \in \mathcal{F}$ and all $xy \in \{00, 01, 10, 11\}$, then $S_{1|\mathcal{F}} = \sum_{F\in\mathcal{F}} S_{1|F}\, P(F)$.*

Observe that this generalises the property in Equation (B21), which comes about if $\mathcal{F}$ is taken to constitute the full range of F.
